# Cystatin 6 (CST6) and Legumain (LGMN) are potential mediators in the pathogenesis of preeclampsia

**DOI:** 10.1038/s41598-025-96823-9

**Published:** 2025-04-15

**Authors:** Stefan M. Botha, Lucy A. Bartho, Sunhild Hartmann, Ping Cannon, Anna Nguyen, Tuong-Vi Nguyen, Natasha Pritchard, Ralf Dechend, Olivia Nonn, Stephen Tong, Tu’uhevaha J. Kaitu’u-Lino

**Affiliations:** 1 Translational Obstetrics Group, The Department of Obstetrics, Gynaecology and Newborn Health, Mercy Hospital for Women, University of Melbourne, Heidelberg, Victoria 3084 Australia; 2 Mercy Perinatal, Mercy Hospital for Women, Heidelberg, Victoria Australia; 3 Charité - Universitätsmedizin Berlin, corporate member of Freie Universität Berlin and Humboldt-Universität zu Berlin, Berlin, Germany; 4 Experimental and Clinical Research Center, a cooperation between Max-Delbrück-Center for Molecular Medicine in the Helmholtz Association and the Charité - Universitätsmedizin Berlin, Berlin, Germany; 5 Max-Delbrück-Center for Molecular Medicine in the Helmholtz Association (MDC), Berlin, Germany; 6 German Center for Cardiovascular Research (DZHK), Partner Site Berlin, Berlin, Germany; 7 Department of Cardiology and Nephrology, HELIOS Clinic, Berlin, Germany

**Keywords:** Cystatin 6, CST6, Legumain, LGMN, Pregnancy, Preeclampsia, Placenta, Trophoblast, Predictive markers, Translational research, Molecular medicine

## Abstract

Preeclampsia results from placental insufficiency and causes maternal endothelial dysfunction and multi-organ damage. Our in-silico analysis identified Cystatin 6 (CST6), a cysteine protease inhibitor, as located on the placental surface where it might be released into maternal circulation. This study aimed to characterise CST6 and one of its high affinity targets, Legumain (LGMN), in preeclampsia and assess its biomarker potential by measuring levels in maternal circulation. Placental *CST6* mRNA expression was significantly increased in 78 pregnancies complicated by early-onset preeclampsia (delivering at < 34 weeks’ gestation) relative to 30 gestation matched controls *(P* < 0.0001). *LGMN* mRNA expression was significantly decreased (*P* = 0.0309). Circulating CST6 was increased in 35 pregnancies complicated by early-onset preeclampsia (< 34 weeks’ gestation) relative to 27 gestation matched controls (*P* = 0.0261), and LGMN levels remained unchanged. At 36 weeks’ gestation, circulating CST6 was significantly increased *(P =* 0.001), while LGMN was significantly decreased (*P* = 0.0135) in 21 pregnancies preceding diagnosis of preeclampsia at term, compared to 184 pregnancies that did not develop preeclampsia. Human trophoblast stem cells (hTSC) were differentiated into syncytiotrophoblast or extravillous trophoblast (EVT) to evaluate CST6 and LGMN expression in these trophoblast lineages. *CST6* and *LGMN* mRNA expression were significantly increased across 96 h after syncytiotrophoblast (*P* = 0.0066 and *P* = 0.0010 respectively) and EVT differentiation *(P =* 0.0618 and *P* = 0.0016 respectively), with the highest expression in syncytiotrophoblast. Computational analysis of two publicly available single-cell and single-nuclei RNA sequencing datasets correlated with the expression pattern observed *in vitro.* When syncytiotrophoblast cells were exposed to hypoxia (1% O_2_ vs. 8% O_2_), *CST6* expression significantly increased (*P* = 0.0079), whilst *LGMN* expression was unchanged. The vascular endothelium may serve as an additional source of circulating CST6 and LGMN in preeclampsia.  Induction of dysfunction in endothelial cells by TNFα, caused reduced *CST6* expression (*P* = 0.0036), whilst *LGMN* expression remained unchanged. Administering recombinant CST6 to endothelial cells enhanced markers of endothelial dysfunction and LGMN expression in the presence of TNFα. These findings indicate an inverse relationship between CST6 and LGMN in the placenta and maternal circulation in preeclampsia. We suggest elevated circulating levels of CST6 may be induced by placental hypoxia. This study provides novel insight into the dysregulation of CST6 and LGMN in preeclampsia and introduces their potential roles in human pregnancy and associated pathology.

**Supplementary Information:**

The online version contains supplementary material available at 10.1038/s41598-025-96823-9.

## Introduction

Preeclampsia is a serious pregnancy complication affecting multiple organs. The disease is characterised by severe hypertension after 20 weeks’ gestation with one or more complications^1^. These complications may include placental insufficiency, proteinuria and compromise of maternal organs^1^. Globally, up to 4 million women are diagnosed with preeclampsia each year, causing over 70,000 maternal deaths, and 500,000 infant deaths annually^2,3^. The only cure is delivery, which may come with severe consequences when preeclampsia strikes early^4^.

It is known that the placenta releases molecules into maternal circulation during pregnancy^5^. In preeclampsia, these molecules may exacerbate endothelial dysfunction and/or maternal organ dysfunction^5,6^. Investigation of the placenta may offer a potential source of novel biomarkers, therapeutic targets, and insights for understanding the pathophysiology of preeclampsia. To date, soluble fms-like tyrosine kinase-1 (sFlt-1) and placental growth factor (PlGF) are the main biomarkers used clinically for ruling out preeclampsia^7^. However, no established biomarkers alone accurately predict the development of preeclampsia.

Cystatins belong to a superfamily of cysteine protease inhibitors categorised into three families according to their structural properties^8,9^. Members of the type 2 cystatin family, including Cystatin 6 (CST6), are primarily secreted and widely distributed throughout bodily fluids and tissues^10^. Family 2 cystatins have attracted significant interest owing to their capacity to inhibit several proteases such as legumain and cathepsins^11–15^. Cystatins play critical roles in endometrial development and placental remodelling across various mammalian species, including sheep, cows, and pigs^16,17^. While CST6 has been reported to contribute to both tumour suppression and growth outside of pregnancy^11,13,18,19^, its role in pregnancy and preeclampsia remains largely unknown.

CST6 has a high binding affinity for Legumain (LGMN), a lysosomal cysteine protease involved in antigen processing, neuronal cell death, and tumour cell invasion^19–24^. CST6 has been shown to regulate LGMN activity and thus may regulate LGMN induced proteolysis^20,25^. Notably, LGMN has been shown to support implantation and placentation in the bovine uterus^17^. These findings highlight the potential that both CST6 and LGMN may exert additional, yet undefined, roles in pregnancy and preeclampsia.

In this study, we aimed to characterise CST6 and LGMN in the placenta and in preeclampsia. Additionally, we explored the regulation of CST6 and LGMN using a human trophoblast stem cell (hTSC) differentiation model^26^. We compared these findings with two publicly available datasets: a single-cell RNA sequencing (scRNA-seq) organoid dataset (GEO accession number: GSE174481^27^), and a single-nuclei RNA sequencing (snRNA-seq) placental dataset (EGA accession number: EGAS00001005681^28^). Finally, we sought to identify the source of circulating CST6 and LGMN and investigate the effects of recombinant CST6 on endothelial dysfunction. 

## Results

### CST6 mRNA expression is increased while LGMN mRNA expression is decreased in placenta from pregnancies complicated by early-onset preeclampsia (< 34 weeks’ gestation)

**Fig. 1:**
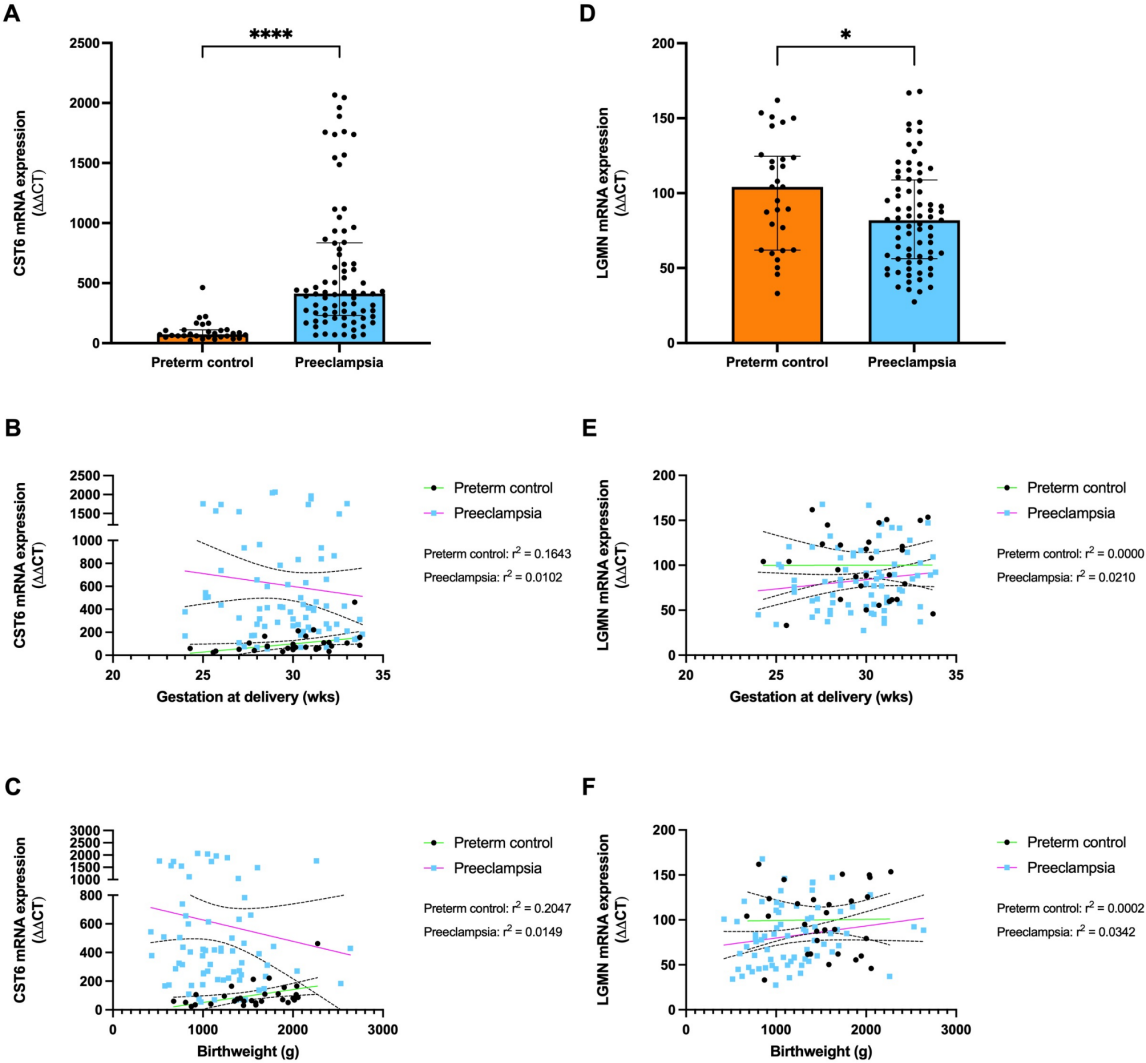
CST6 mRNA expression increased while LGMN mRNA expression is reduced in placental lysates from patients with early‑onset preeclampsia (< 34 weeks’ gestation). (**A**) CST6 mRNA expression was examined via RT-qPCR relative to gestation-matched controls (*n* = 30, orange bar), in placentas obtained from patients with early-onset preeclampsia (*n* = 78, blue bar), CST6 expression was significantly increased, with a significant association with (**B**) gestational age at collection (green line, r^2^ = 0.1643, *p* = 0.0263) and (**C**) fetal birthweight (green line, r^2^ = 0.2047, *p* = 0.0121) in the control group. In the preeclampsia group there was no association between CST6 expression and (**B**) gestational age at collection (pink line, r^2^ = 0.0102, *p* = 0.2900) and (**C**) fetal birthweight (pink line, r^2^ = 0.0149, *p* = 0.7823) (**D**) LGMN mRNA expression was examined via RT-qPCR relative to gestation-matched controls (*n* = 30, orange bar), in placentas obtained from patients with early-onset preeclampsia (*n* = 78, blue bar), LGMN expression was significantly reduced, with no significant association with (**E**) gestational age at collection (green line, r^2^ = 0.0000, *p* = 0.9924) and (**F**) fetal birthweight (green line, r^2^ = 0.0002, *p* = 0.9382) in the control group. The same was observed in the preeclampsia group between (**E**) gestational age at collection (green line, r^2^ = 0.0210, *p* = 0.2218) and (**F**) fetal birthweight (green line, r^2^ = 0.0342, *P* = 0.1197). Each dot represents individual participants. The significance of the data was determined using a Mann–Whitney U test and a simple linear regression was used to determine the association of CST6 and LGMN expression/concentration with fetal birthweight and gestation age at delivery. Data is expressed as median ± interquartile range. **** *p* < 0.0001, * *p* < 0.05.

To assess whether *CST6* and *LGMN* mRNA expression is dysregulated in placentas from 78 women with early-onset preeclampsia, *CST6* and *LGMN* expression was measured in placental lysates compared to 30 gestation-matched controls. *CST6* expression was significantly increased 5.65-fold in placenta from patients with early-onset preeclampsia, relative to controls (Fig. 1A, *P* < 0.0001). Additional analyses of *CST6* mRNA expression showed a significant but weak to moderate association in the control group with gestational age at delivery (Fig. 1B, green line, r^2^ = 0.1643, *P* = 0.0263) and fetal birth weight in the control group (Fig. 1C, green line, r^2^ = 0.2047, *P* = 0.0121). *CST6* mRNA expression increased with gestational age and with increasing fetal birth weight in the control group. No association with gestational age at delivery (Fig. 1B, pink line, r^2^ = 0.0102, *P* = 0.2900), fetal birth weight (Fig. 1C, pink line, r^2^ = 0.0149, *P* = 0.7823) nor fetal sex (data not shown) was observed in the preeclampsia group. CST6 had no significant association with fetal sex (data not shown).

*LGMN* expression was significantly reduced in placenta from patients with early-onset preeclampsia, relative to controls (Fig. 1D, *P* = 0.0309). Additional analysis of *LGMN* expression showed no significant association with gestational age at delivery in the control (Fig. 1E, green line, r^2^ = 0.0000, *P* = 0.9924) and preeclampsia groups (Fig. 1E, pink line, r^2^ = 0.0210, *P* = 0.2218). The same was observed regarding fetal birth weight for the control (Fig. 1F, green line, r^2^ = 0.0002, *P* = 0.9382) and preeclampsia (Fig. 1F, pink line, r^2^ = 0.0342, *P* = 0.1197) groups. Unlike CST6, *LGMN* mRNA expression increased with gestational age and with increasing fetal birth weight in the preeclampsia group. No significant association with fetal sex (data not shown) was observed for either group.

Thus, significantly increased *CST6* mRNA expression is accompanied by significantly reduced *LGMN* mRNA expression in placentas from women with early-onset preeclampsia.

### Circulating CST6 is elevated in pregnancies complicated by early-onset preeclampsia

**Fig. 2:**
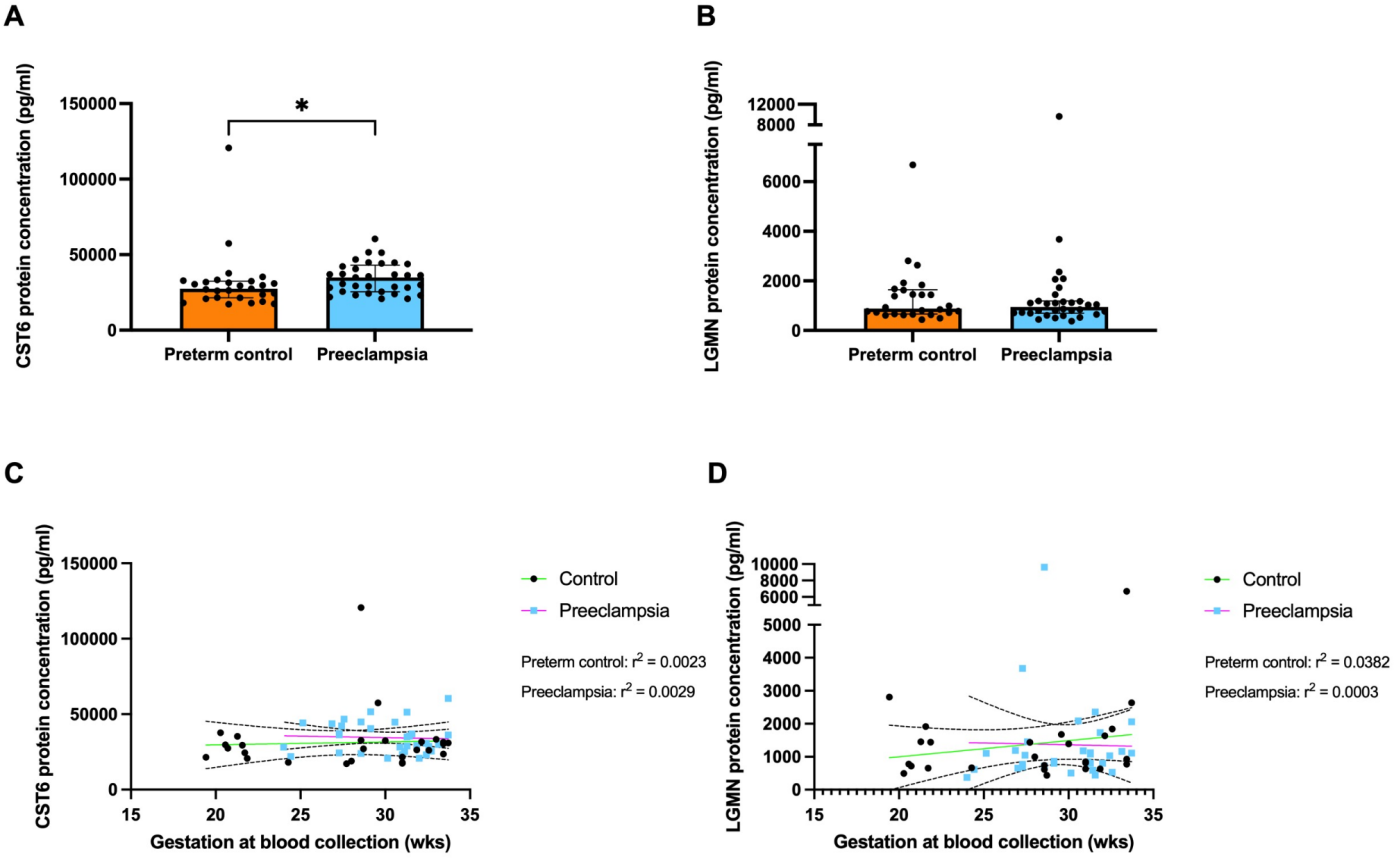
CST6 protein concentration is increased while LGMN protein concentration in plasma is unchanged in patients with early-onset preeclampsia (< 34 weeks’ gestation). (**A**) CST6 protein concentration was measured via ELISA relative to gestation-matched controls (*n* = 27, orange bar), in placentas obtained from patients with early-onset preeclampsia (*n* = 35, blue bar). (**B**) LGMN protein concentration was measured in the same cohort, and no change in protein concentration was observed. (**C**) CST6 levels were significantly increased with no significant association with gestational age at collection for both the control (r^2^ = 0.0023, *p* = 0.8131) and preeclampsia (r^2^ = 0.0029, *p* = 0.7676) groups. (**D**) There was no significant association between LGMN protein concentration and gestational age at collection for both the control (r^2^ = 0.0382, *p* = 0.3387) and preeclampsia (r^2^ = 0.0003, *p* = 0.9228) groups. Each dot represents individual participants. The significance of the data was determined by a Mann–Whitney U test and a simple linear regression was used to determine the association of CST6 and LGMN expression/concentration with gestation at blood collection. Data is expressed as median ± interquartile range. * *p* < 0.05.

Following confirmation of dysregulated CST6 and LGMN expression in the placenta of women with early-onset preeclampsia, we next sought to investigate circulating levels of CST6 and LGMN. CST6 protein concentration was significantly increased in plasma obtained from 35 pregnancies complicated by early-onset preeclampsia at a median of 3.50 × 10^4^ pg/mL (IQR, 2.55 × 10^4^ pg/mL – 6.05 × 10^4^ pg/mL) compared to 27 gestation-matched controls at a median of 2.74 × 10^4^ pg/mL (IQR, 1.72 × 10^4^ pg/mL – 1.21 × 10^5^ pg/mL) (Fig. 2A, *P* = 0.0108). In this same cohort, LGMN levels were unchanged in women with preeclampsia (Fig. 2B). The median for LGMN in the preeclampsia group was 9.48 × 10^2^ pg/mL (IQR, 3.73 × 10^2^ pg/mL – 9.62 × 10^3^ pg/mL) compared to the gestation-matched control median of 8.88 × 10^2^ pg/mL (IQR, 4.39 × 10^2^ pg/mL – 6.67 × 10^3^ pg/mL). Further analysis of CST6 protein concentration showed no significant association with gestational age at sample collection (Fig. 2C) for either the control (green line, r^2^ = 0.0023, *P* = 0.8131) or preeclampsia groups (pink line, r^2^ = 0.0029, *P* = 0.7676). The same was observed for LGMN (Fig. 2D) in the control (green line, r^2^ = 0.0382, *P* = 0.3387) and preeclampsia (pink line, r^2^ = 0.0003, *P* = 0.9228) group. No association was observed for both molecules with fetal sex (data not shown). The increase in CST6 in maternal plasma correlates with the elevated placental mRNA expression observed in women with early-onset preeclampsia.

### Circulating CST6 is elevated whilst LGMN is decreased at 36 weeks’ gestation, preceding diagnosis of term preeclampsia

**Fig. 3:**
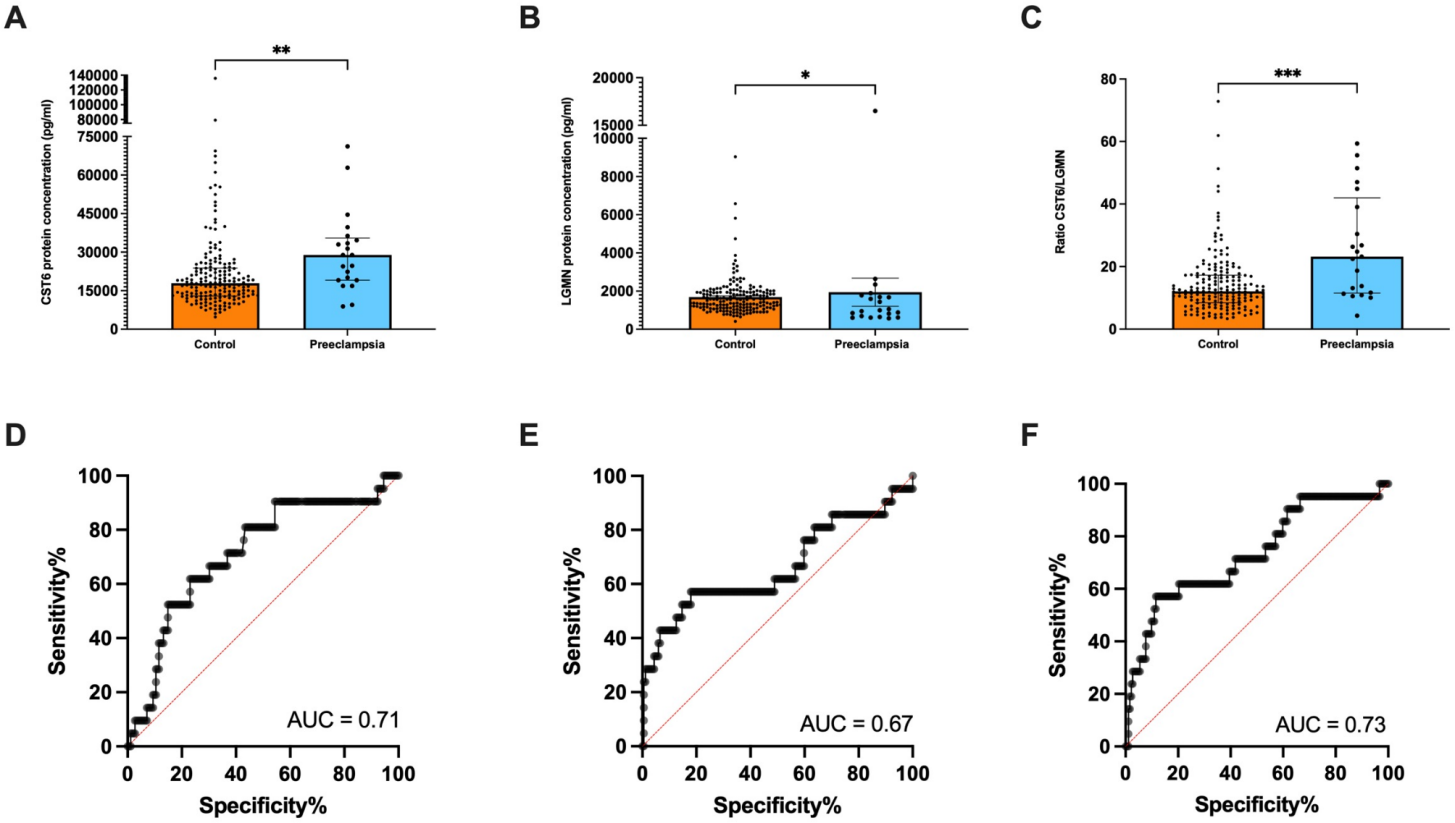
CST6 protein concentration was increased while LGMN protein concentration was decreased in patients preceding diagnosis of preeclampsia (36 weeks’ gestation). (**A**) In the BUMPS cohort, circulating CST6 was increased in women who developed preeclampsia at term (*n* = 21) compared to gestation-matched controls (*n* = 184). (**B**) In the same cohort, circulating LGMN was decreased in women who developed preeclampsia at term compared to gestation-matched controls. (**C**) The ratio of CST6/LGMN was plotted and, in the same cohort, was increased in women who developed preeclampsia at term compared to gestation-matched controls. Area under the receiver operator curve (AUC) of (**D**) 0.71, (**E**) 0.67 and (**F**) 0.73. Each dot represents individual participants. The significance of the data was determined using a Mann–Whitney U test. Data is presented as median ± interquartile range. *** *P* < 0.001, ** *P* < 0.01, * *P* < 0.05.

To assess the predictive potential of CST6 and LGMN, we measured circulating CST6 and LGMN in the BUMPS cohort of samples collected around 36 weeks’ gestation. This cohort consisted of an unbiased sample population including 21 women who later developed term preeclampsia and 184 gestation-matched controls who did not develop preeclampsia. CST6 protein concentration was significantly increased in patients preceding diagnosis of term preeclampsia with a median of 2.47 × 10^4^ pg/mL (IQR, 8.87 × 10^3^ pg/mL – 6.23 × 10^4^ pg/mL) (Fig. 3A, *P* = 0.008), compared to gestation-matched controls, median of 1.80 × 10^4^ pg/mL (IQR, 4.79 × 10^3^ pg/mL – 1.36 × 10^5^ pg/mL). LGMN protein concentration was significantly decreased in patients preceding diagnosis of term preeclampsia, median of 9.96 × 10^2^ pg/mL (IQR, 5.73 × 10^2^ pg/mL – 1.65 × 10^4^ pg/mL) (Fig. 3B, *P* = 0.0135), compared to gestation-matched controls, median of 1.50 × 10^3^ pg/mL (IQR, 4.10 × 10^2^ pg/mL – 9.03 × 10^3^ pg/mL). We also assessed the predictive power of the two molecules as a ratio (Fig. 3C). There was a significant increase in the CST6/LGMN ratio in patients who went on to develop preeclampsia (23.19 [IQR, 4.31–59.34], *P* = 0.0003), compared to those delivering at term without preeclampsia (12.09 [IQR, 3.50–72.87]).

Further analysis using the area under the receiver operating characteristic curve (AUC) was conducted to assess the discriminatory power of circulating CST6, LGMN and CST6/LGMN ratio at 36 weeks’ gestation. The test showed modest performance for CST6 (Figure 3D, AUC = 0.71). For LGMN, there was also modest discrimination (Figure 3E, AUC = 0.67). The AUC of the ratio of CST6/LGMN was 0.73 (Figure 3F). Thus, while we confirm dysregulated circulating CST6 and LGMN preceding diagnosis of term preeclampsia, the AUC for either molecule alone, or as a ratio, were modest.

### CST6 and LGMN are expressed in syncytiotrophoblast and EVT cells following differentiation

Given that CST6 and LGMN are dysregulated in the placenta, a hTSC cell differentiation model was used^26^ to determine CST6 and LGMN expression in various trophoblast stem cells lines. Trophoblast cells were differentiated into extravillous trophoblasts (EVTs) or syncytiotrophoblasts, the two main types of differentiated trophoblast lineages.

**Fig. 4:**
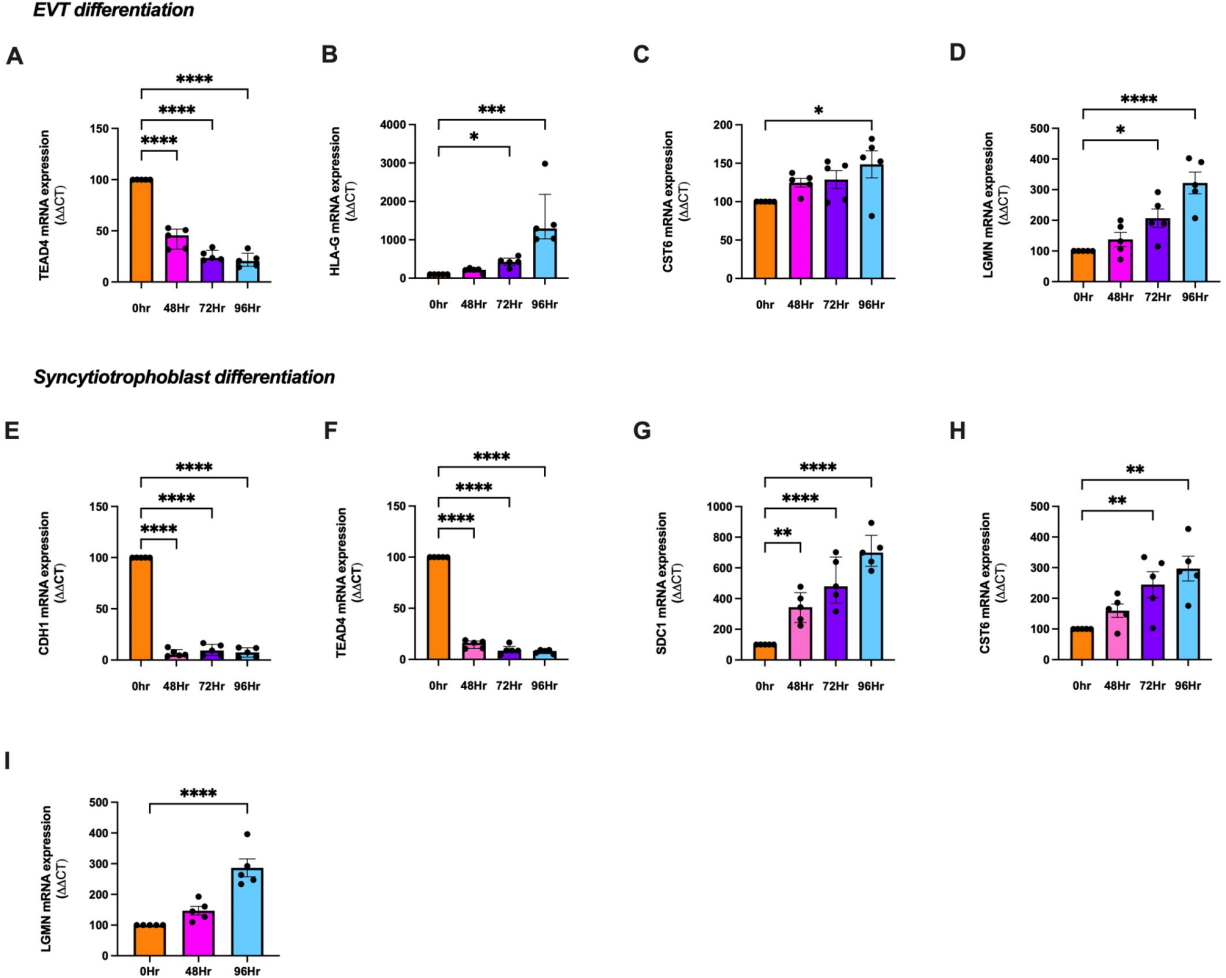
CST6 and LGMN expression with differentiation of hTSCs to EVT and syncytiotrophoblasts. hTSCs were differentiated to EVT or syncytiotrophoblasts and measured across 0, 48, 72 and 96 h. mRNA expression was assessed via qRT-PCR. EVT differentiation was confirmed by a significant loss in TEAD4 (**A**) and a significant gain of HLA-G (**B**). (**D**) Following differentiation of hTSCs into EVTs, (**C**) CST6 and (**D**) LGMN mRNA expression was significantly increased across 96 h. Syncytiotrophoblast differentiation was confirmed by a significant loss of CDH2 (**E**), significant loss of TEAD4 (**F**) and significant gain of SDC1 (**G**). Following hTSC differentiation into syncytiotrophoblast cells, (**H**) CST6 and (**I**) LGMN mRNA expression was significantly increased across 96 h. mRNA expression was normalised to the geometric mean of housekeeper genes. To calculate significance, one-way ANOVA (parametric) or a Kruskal Wallis test (non-parametric) was used. Data expressed as mean ± SEM for experiments (A, D, E, F, H and I) or median ± IQR (B, C and G). Experiments repeated *n* = 5 in triplicate. **** *p* < 0.0001, *** *p* < 0.001, ** *p* < 0.01, *p < 0.05.

Successful differentiation of hTSCs into EVTs was confirmed through a significant loss of cytotrophoblast progenitor marker *TEAD4* (Fig. 4A, *P* < 0.0001 across 96 h) and a significant increase in established EVT marker, *HLA-G* (Fig. 4B, *P* = 0.0264 at 72 h and *P* < 0.001 at 96 h). *CST6* expression was significantly increased with EVT differentiation (Fig. 4C, *P* = 0.0219 across 96 h). *LGMN* expression was also significantly increased with EVT differentiation (Fig. 4D, *P* = 0.0267 at 72 h and *P* < 0.001 at 96 h).

Successful differentiation and fusion of hTSCs into syncytiotrophoblast cells was confirmed by a significant loss of cell surface marker, *CDH2* (Fig. 4E, *P* < 0.0001 across 96 h) and cytotrophoblast progenitor marker, *TEAD4* (Fig. 4F, *P* < 0.0001 across 96 h) as well as significantly increased syncytiotrophoblast marker, *SDC1* (Fig. 4G, *P* < 0.0001 across 96 h). *CST6* expression was significantly increased as hTSCs differentiated into syncytialised cells (Fig. 4H, *P* = 0.0095 at 2 h and *P* = 0.0012 at 96 h). In conjunction, *LGMN* expression was also significantly increased as hTSCs differentiated into syncytialised cells (Fig. 4I, *P* < 0.0001 at 96 h).

Taken together, this confirmed principal expression of CST6 and LGMN in syncytiotrophoblast cells when compared to other cell trophoblast lineages.

### scRNA-seq analysis determines CST6 and LGMN expression in an organoid model of trophoblast differentiation

We next sought to validate our in vitro and in vivo observations in a publicly available scRNA-seq dataset (GEO accession number: GSE174481). This dataset was established using three-dimensional hTSC-derived organoids (*n* = 3) that were either cultured as hTSCs or induced to differentiate into EVTs^27^.

**Fig. 5:**
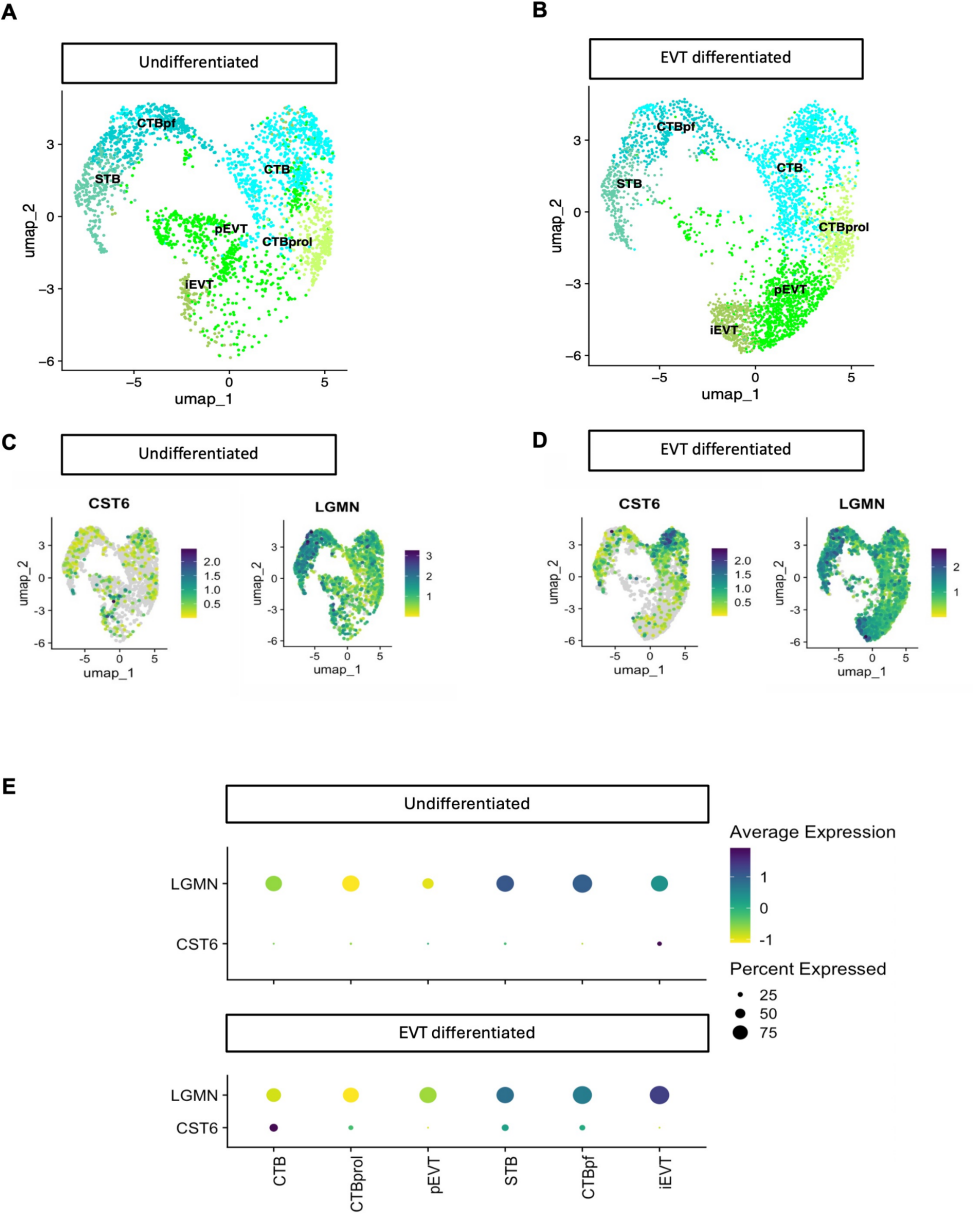
Single cell RNA sequencing analysis of CST6 and LGMN in hTSC organoids. Transcriptomic analysis of a publicly available scRNA-seq dataset of three-dimensional hTSC-derived organoids (*n* = 3) treated under hTSC conditions or induced to differentiate to EVTs across 21 days (GEO accession number GSE174481^27^). Analysis resulted in 6 distinct transcriptomic cell identities: cytotrophoblast (CTB), proliferative cytotrophoblast (CTBprol), pre-fusion cytotrophoblast (CTBpf), progenitor EVT (pEVT), invasive EVT (iEVT) and syncytiotrophoblast (STB). UMAP plot of cell identities in (**A**) undifferentiated and (**B**) EVT differentiated hTSC organoids. (**C**) UMAP plot of CST6 and LGMN expression relative to cell identities in undifferentiated organoids. (**D**) UMAP plot of CST6 and LGMN expression relative to cell identities in EVT differentiated organoids. (**E**) Dot plot of CST6 and LGMN to indicate levels of gene expression in cell proportions of specific cell identities for each condition.

6,354 high-quality cells were obtained from the single-cell RNA-sequencing dataset following quality control measures. Uniform Manifold Approximation and Projection (UMAP) was employed to visualise the relationships between these cells based on their gene expression profiles as previously described (Fig. 5A, B, C and D)^29,30^. Six distinct cell subtypes were identified within these clusters and assigned labels (Fig. 5A and E): cytotrophoblast (CTB), proliferative cytotrophoblast (CTBprol), pre-fusion cytotrophoblast (CTBpf), progenitor EVT (pEVT), invasive EVT (iEVT) and syncytiotrophoblast (STB). A population of cells within both undifferentiated and differentiated organoid cultures exhibited transcriptional characteristics resembling syncytiotrophoblasts (STB). Supplementary Fig. 1 depicts a dot plot of markers of known trophoblast genes identifying each cluster^27^.

Our analysis showed low expression of *LGMN* and *CST6* within cytotrophoblast cell identities (Fig. 5E). *LGMN* expression was identified mostly in the STB and CTBpf cell populations (~ 75% of total population within both undifferentiated and EVT differentiated organoid cultures). *LGMN* was expressed in 75% of the total iEVT cell population in the undifferentiated and EVT differentiated organoids (Fig. 5E). *CST6* was found to be lowly expressed in the iEVT population in EVT differentiated organoids. The expression pattern observed depicts modest correlation with our observations in vitro.

### snRNA-seq analysis determines cell type specific CST6 and LGMN mRNA expression in placenta

Using snRNA-seq analysis, we conducted an additional validation of specific cell type expression of CST6 and LGMN in the placenta of both early-onset preeclampsia and controls. This analysis was adapted from the data, analysis and methodology outlined in the manuscript by Nonn, et al. (EGA accession number: EGAS00001005681 and processed data from original manuscript available at: https://zenodo.org/records/8159511 upon request)^28^.

**Fig. 6:**
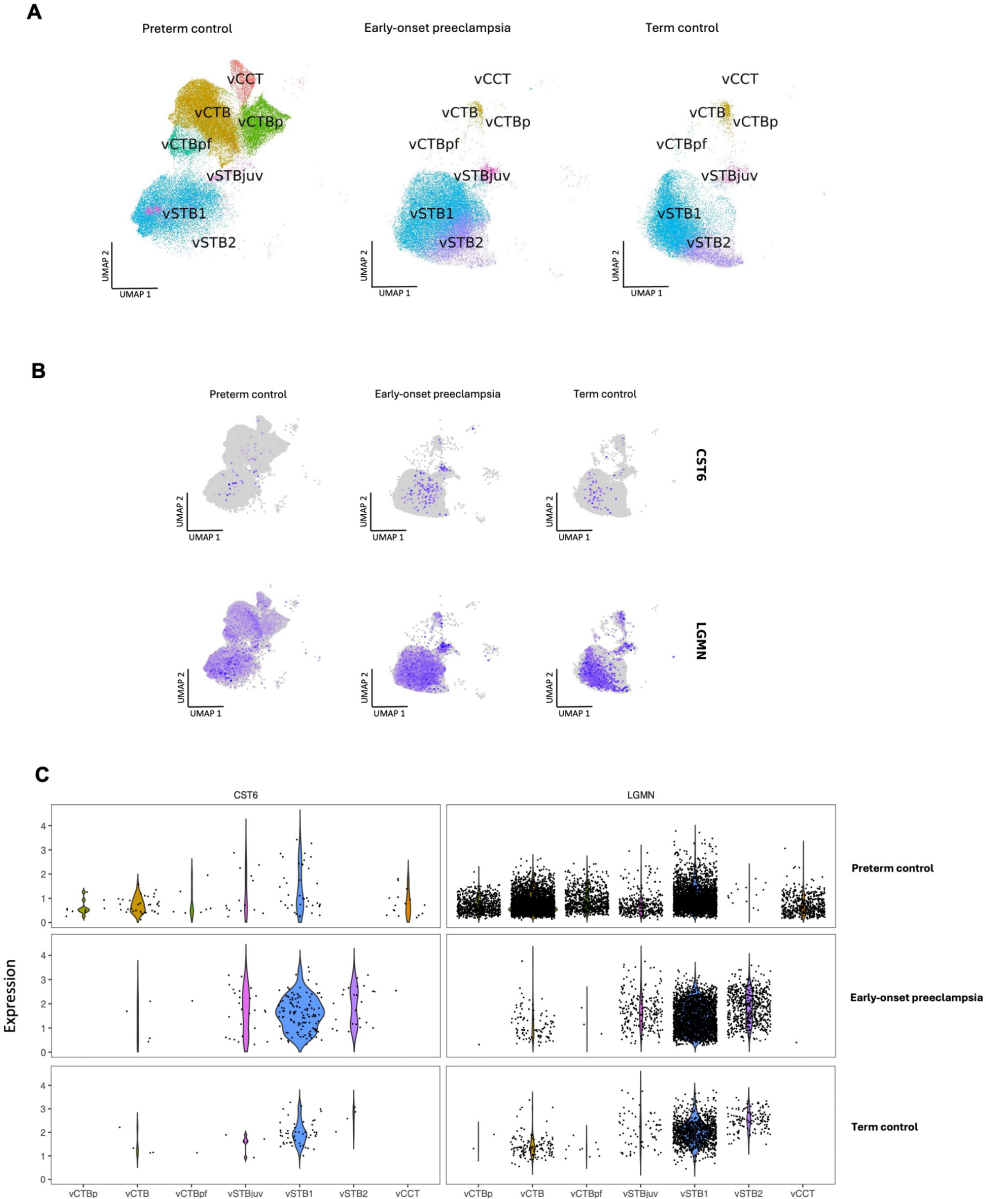
Single-nuclei RNA sequencing (snRNA-seq) analysis of early-onset preeclamptic, healthy and preterm placenta. (**A**) and (**B**), Uniform manifold approximation and projection (UMAP) visualising and analysis resulted in 7 distinct transcriptomic cell identities obtained from the data: villous syncytiotrophoblast (vSTB1 and vSTB2), villous juvenile syncytiotrophoblast (vSTBjuv), villous pre-fusion cytotrophoblast (vCTBpf), villous proliferative cytotrophoblast (vCTBp), villous cytotrophoblast (vCTB) and villous cell column trophoblast (vCCT). Samples from preterm pregnancy, early-onset preeclampsia and term healthy pregnancy were integrated for visualisation. Each dot represents a nucleus with each colour representing a cell identity. (**C**) Violin plot of CST6 and LGMN to indicate levels of gene expression in cell proportions of specific cell identities for each condition. Code available at https://github.com/HiDiHlabs/preeclamspsia_Nonn_etal/, data available in supplementary methods of manuscript^28^.

We utilised UMAP analysis to visualise the relationships between cell identifies based on their gene expression profiles (Fig. 6A and B). Furthermore, we used this analysis to visualise the expression of CST6 and LGMN within specific cell identities (Fig. 6B). Of the 15 villous cell types and cell states of that were identified in the original dataset^28^, we narrowed our analysis to focus on 7 trophoblast cell identifies and states (Fig. 6A). These include villous syncytiotrophoblast (vSTB1 and vSTB2), villous juvenile syncytiotrophoblast (vSTBjuv), villous pre-fusion cytotrophoblast (vCTBpf), villous proliferative cytotrophoblast (vCTBp), villous cytotrophoblast (vCTB) and villous cell column trophoblast (vCCT) which are representative of an early EVT cell identity. Notable variations in cell type composition within trophoblast cell identities in placental samples were observed between the different groups (Fig. 6A).

We next sought to compare the specific gene expression profile of early-onset preeclampsia (27–33 weeks’ gestation) to preterm placental samples from elective pregnancy terminations (5–10 weeks’ gestation) and to term control placental samples (38–40 weeks’ gestation) (Fig. 6C). We used a violin plot to visualise the expression patterns of CST6 and LGMN within cell identities between the groups (Fig. 6C).

From our analysis we observed a similar overall expression profile to what was observed in our RT-qPCR analysis of placental tissue samples. We observed a modest increase in *CST6* mRNA expression in early-onset preeclampsia compared to preterm controls (Fig. 6C). For LGMN, we observed a decrease in *LGMN* mRNA expression in early-onset preeclampsia compared to preterm controls (Fig. 6C). This is marked by a reduction of specific placental cell identities between preterm controls and the remaining two groups. We observed a reduction in both *CST6* and *LGMN* mRNA expression in healthy term placenta when compared to early-onset preeclamptic placenta (Fig. 6C).

There was low expression of *CST6* in cytotrophoblast cell populations in all cohorts (Fig. 6C). *CST6* mRNA expression was localised to vSTB cell populations across all groups, with expression being consistent to the vSTB1 cell identity. *LGMN* mRNA was expressed in all cell identities in the preterm control cohort (Fig. 6C). Additionally, *LGMN* expression was localised to the vCTB and vSTB cell identities. There was considerable *LGMN* mRNA expression in the vCCT cell population in the preterm control cohort (Fig. 6C). Overall *LGMN* mRNA expression was reduced within all cell populations in both early-onset preeclampsia and term controls compared to the preterm controls. Here, *LGMN* mRNA expression was localised to vSTB populations, mainly vSTB1 and vSTB2 cell identities. There was modest expression of *LGMN* in vCTB expression in early-onset preeclampsia and term control groups.

The expression pattern observed in this analysis correlates with our observations in placental tissue and in vitro. Furthermore, we see some concordance with our results from our scRNA-seq analysis of the hTSC trophoblast organoid dataset.

### CST6 is increased in syncytiotrophoblast cells exposed to hypoxia while LGMN remains unchanged

**Fig. 7:**
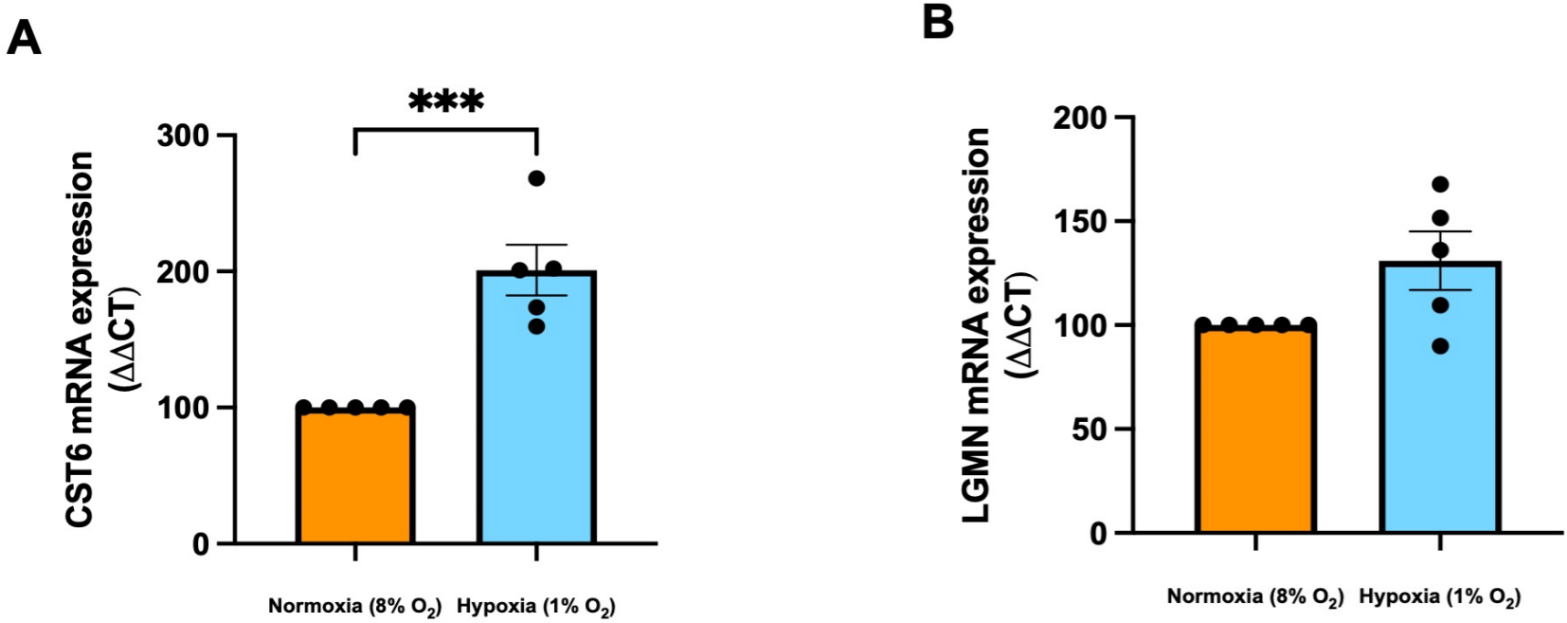
Regulation of CST6 and LGMN expression in syncytiotrophoblast cells. (**A**) CST6 expression was significantly increased in syncytiotrophoblast cells incubated in a hypoxic environment (1% O_2_) compared to control normoxic environment (8% O_2_). (**B**) LGMN expression was unchanged when incubated in a hypoxic environment relatively to control. mRNA expression was normalised to the geometric mean of housekeeper genes or single housekeeping gene depending on the treatment. To calculate significance, one-way ANOVA or Unpaired t test (parametric) or a Kruskal Wallis test (non-parametric) was used. Data expressed as mean ± SEM for experiments. Experiments repeated *n* = 5 in triplicate. *** *p* < 0.001.

Preeclampsia is associated with inflammation and hypoxia. Therefore, following confirmation of the placental cell type expressing *CST6* and *LGMN*, we next assessed the regulation of these molecules in syncytiotrophoblast exposed to increasing doses of pro-inflammatory cytokines, TNFα and IL-6, and hypoxia (1% O_2_). *CST6* mRNA expression was unchanged in syncytiotrophoblast cells exposed to inflammatory cytokines IL-6 and TNFα (supplementary Fig. 2A and supplementary Fig. 2B). *LGMN* mRNA expression was also unchanged in syncytiotrophoblast cells exposed to inflammatory cytokines IL-6 and TNFα (supplementary Fig. 2C and supplementary Fig. 2D). *CST6* expression was significantly increased in syncytiotrophoblast cells incubated in hypoxia (1% O_2_) (Fig. 7A, *P* = 0.0006) relative to cells incubated at 8% O_2_. *LGMN* expression was unchanged in syncytiotrophoblast cells incubated in hypoxia (1% O_2_) relative to controls (Fig. 7B).

### CST6 and LGMN expression is unchanged in placental explants exposed to inflammation and hypoxia

We next assessed the regulation of *CST6* and *LGMN* in placental explants, isolated from term placenta, exposed to inflammatory cytokines, TNFα (*n* = 5) and IL-6 (*n* = 5), and hypoxia (1% O_2_) (*n* = 5). *CST6* expression was unchanged when treated with IL-6 (Supplementary Fig. 3A) and TNFα (supplementary Fig. 3B) at 10ng/ml relative to control. The same was observed in explants exposed to hypoxia (supplementary Fig. 3C). *LGMN* expression was unchanged when treated with IL-6 (Supplementary Fig. 3D) and TNFα (supplementary Fig. 3E) at 10ng/ml relative to control. The same was observed in explants exposed to hypoxia (supplementary Fig. 3F).

### Endothelial dysfunction downregulates CST6 expression and has no effect on LGMN

**Fig. 8:**
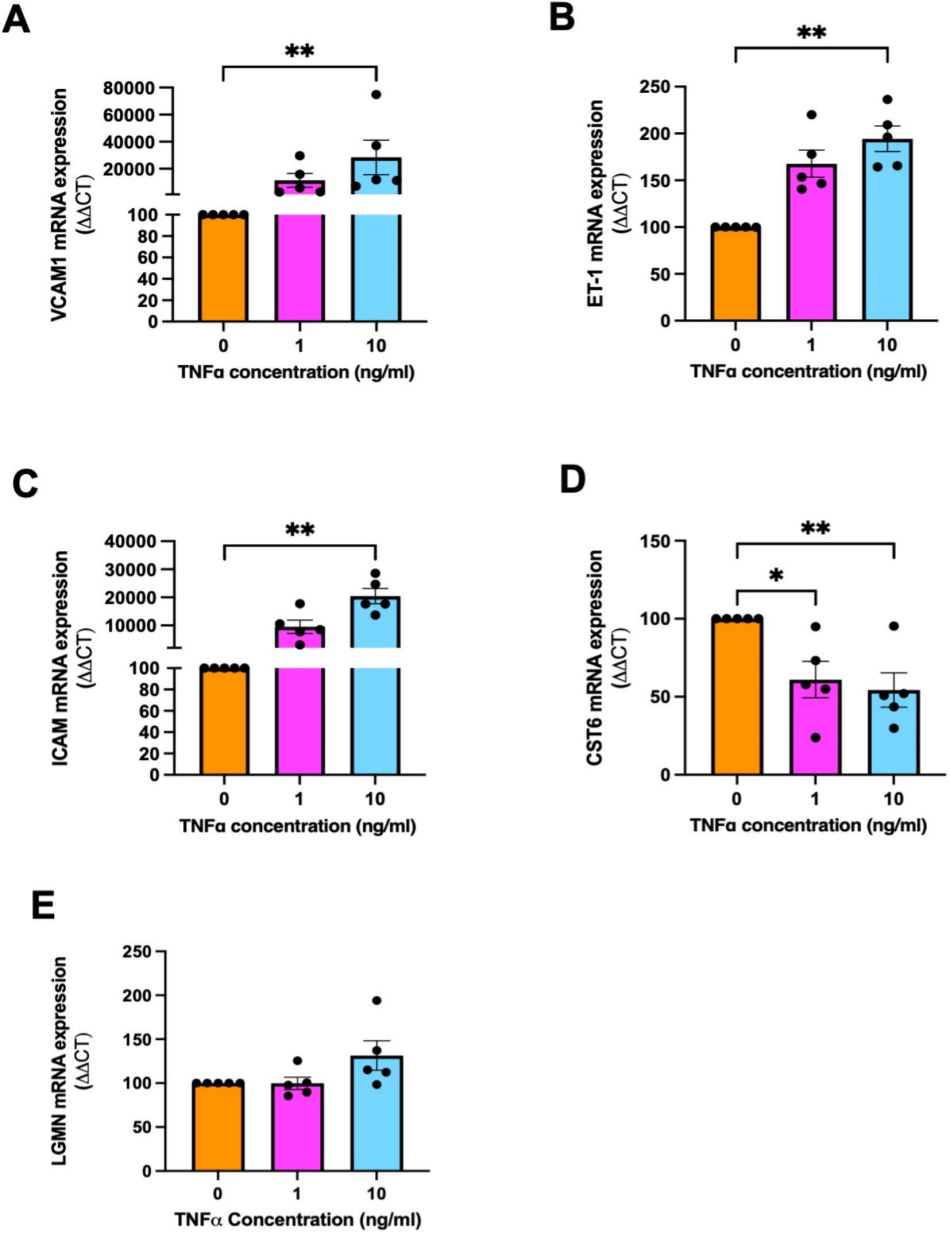
Induced endothelial dysfunction in HUVECs has no effect on LGMN expression but decreases CST6 expression. Successful induction of endothelial dysfunction in HUVECs was confirmed by significant increase of: (**A**) VCAM1 (**B**) ET-1 and (**C**) ICAM1. (**D**) CST6 expression in HUVECs was significantly decreased following treatment with increased doses of TNFα. (**E**) LGMN mRNA expression was unchanged. mRNA expression was normalised to the appropriate housekeeping gene. To calculate significance, Kruskal Wallis test was used. Data expressed as mean ± SEM. Experiments repeated *n* = 5 in duplicate. ** *p* < 0.01, *p < 0.05.

Preeclampsia is linked to endothelial dysfunction. Therefore, we measured *CST6* and *LGMN* expression in HUVECs where dysfunction was induced by TNFα. We confirmed successful induction of endothelial dysfunction by confirming the upregulation of *VCAM1* (Fig. 8A, *P* = 0.0031 at 10 ng/mL TNFα), *ET-1* (Fig. 8B, *P* = 0.0034 at 10 ng/mL TNFα) and *ICAM1* (Fig. 8C, *P* = 0.0014 at 10 ng/mL TNFα). CST6 mRNA expression was significantly decreased with increasing concentrations of TNFα (Fig. 8D, *P* = 0.0015) while there was no change in *LGMN* (Fig. 8E). These findings suggest that the elevated levels of circulating CST6 in preeclampsia may not come from dysfunctional endothelium.

### Recombinant CST6 exacerbates endothelial dysfunction but has no effect on LGMN expression in HUVECs

**Fig. 9:**
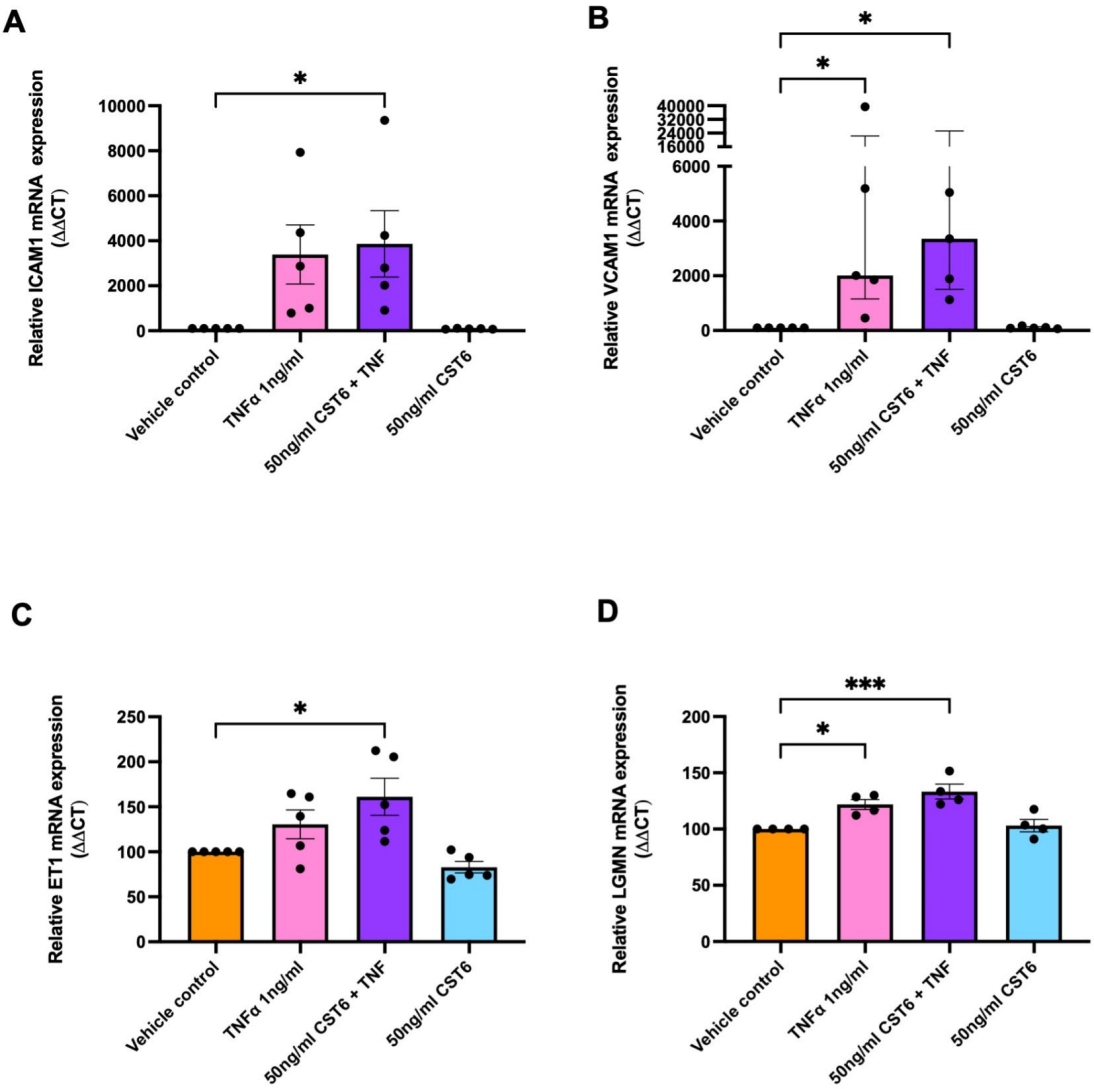
Recombinant CST6 enhances induction of endothelial dysfunction and LGMN in HUVECs. Induction of endothelial dysfunction in HUVECs was assessed by induction of markers: ICAM1 (**A**), VCAM1 (**B**) and ET-1 (**C**). Recombinant CST6 enhanced induction of dysfunction markers and (**D**) LGMN in HUVECs under inflammatory conditions. mRNA expression was normalised to the appropriate housekeeping gene. To calculate significance, one-way ANOVA (parametric) or a Kruskal Wallis test (non-parametric) was used. Data expressed as mean ± SEM for experiments (A, C and D) or median ± IQR (B). Experiments repeated *n* = 5 in duplicate. *** *p* < 0.001, * *p* < 0.05.

As CST6 is increased in maternal circulation in preeclampsia, we exposed HUVEC cells to recombinant CST6. We sought to assess the effects of recombinant CST6 on normal and dysfunctional endothelial cells, and to assess *LGMN* mRNA expression within these cells. The induction of endothelial dysfunction markers *ICAM1* (Fig. 9A, trending - *P* = 0.0782), *VCAM1* (Fig. 9B, *P* = 0.0397) and *ET-1* (Fig. 9C, trending – *P* = 0.2869) by low dose TNFα was more variable than prior experiments, however we confirmed no effect of recombinant CST6 on ICAM*1* (Fig. 9A), *VCAM1* (Fig. 9B) or *ET-1* (Fig. 9C).

When combined with TNFα, recombinant CST6 significantly increased *ICAM1* (Fig. 9A, *P* = 0.0406), *VCAM1* (Fig. 9B, *P* = 0.0249) and *ET-*1 (Fig. 9C, *P* = 0.0144) relative to control.

Recombinant CST6 had similar effects on *LGMN* (Fig. 9D), with recombinant CST6 alone (50ng/mL) having no effect on *LGMN* expression. However, HUVEC treatment with TNFα led to a significant increase in *LGMN* expression (*P* = 0.0189). Furthermore, combining CST6 (50 ng/mL) with TNFα, led to increased *LGMN* expression (Fig. 9D, *P* = 0.0010). Taken together, recombinant CST6 may further amplify endothelial dysfunction in the presence of inflammation.

## Discussion

In this study, we sought to characterise CST6 and LGMN in preeclampsia. We confirmed that CST6 and LGMN are significantly dysregulated in both placenta and circulation in women with preeclampsia, compared to controls. Our findings indicate an inverse relationship between CST6 and LGMN in preeclampsia. CST6 expression and levels in circulation are increased in preeclampsia whilst overall, LGMN is decreased relative to gestation matched controls. The performance of CST6 and LGMN either alone, or in combination as biomarkers for preeclampsia prediction after 36 weeks’ gestation was modest. Whether these molecules are deranged preceding preterm disease diagnosis requires further investigation. This could be measured in an appropriate first trimester cohort.

We confirmed expression of CST6 and LGMN in syncytiotrophoblast and EVT cell lineages. Our snRNA-seq analysis revealed similar expression profiles for both CST6 and LGMN between early-onset preeclampsia and term controls. The similarities likely reflect the third-trimester origin of the placental samples from these cohorts. It is known that the placenta undergoes significant changes throughout gestation, particularly in trophoblast composition, which can influence the expression of specific molecules^31–34^. During the first trimester, the placenta is enriched in proliferative cytotrophoblasts and invasive EVTs essential for implantation and spiral artery remodelling^31,34,35^. In contrast, the second and third trimesters are characterized by an increase in differentiated syncytiotrophoblasts, which facilitate nutrient exchange and hormone production^31,34,35^. These dynamic shifts in cellular composition are associated with stage-specific gene expression profiles^32,33^. Therefore, while first-trimester tissue provides valuable insight into early placental biology, it is important to consider these developmental differences when comparing to later gestational stages. An additional cohort of placenta from late-onset preeclamptic pregnancies would be an appropriate comparison to include for this analysis.

Indeed, alterations in cell populations associated with placental insufficiency may contribute to the dysregulated levels we observed in preeclamptic placenta. With the advent of single cell sequencing technologies, future studies may be able to further interrogate how the expression of these molecules change across gestation within the various cell types.

Proteases are key enzymes involved in protein degradation, protein modification, and extracellular matrix remodelling^36^. They are known to play a role in regulating trophoblast invasion and function^37,38^. CST6 is a potent inhibitor of several cysteine proteases and plays a key role in regulating the proteolytic activity of LGMN^11,39^. LGMN is a cysteine protease that is mainly localised to the endo-lysosomal system^39^. Regulation of LGMN by CST6 safeguards against excessive proteolysis^19,20^. LGMN is involved in a variety of cellular functions beyond placenta, including immune response^40^, neuronal degeneration^41^, cancer progression^19,42^, and tissue remodelling^43^. Prior reports suggest a role for LGMN in regulating autophagy, a cellular process that has been reported as dysfunctional in preeclampsia^44,45^. Furthermore, studies have shown that LGMN deficiency, which may cause lysosomal storage dysfunction, can lead to premature senescence and age-related renal fibrosis^46,47^. This is an important consideration as preeclampsia is also associated with a senescence associated secretory phenotype in the placenta^28,48^.

Although little is known about the function of CST6, there have been several studies on closely related Cystatin C, which is also a cystatin type 2 family member^49–52^. Elevated serum cystatin C levels in early pregnancy are associated with an increased risk of developing preeclampsia^50–52^. Furthermore, several studies have highlighted cystatin C as a potential biomarker of renal dysfunction in preeclampsia^51–55^. Importantly, Cystatin C has been shown to interact with LGMN^36^. Given their close relationship, this may suggest elevated levels of CST6 in preeclampsia could contribute to the development and progression of preeclampsia via similar mechanisms to that of Cystatin C. However, these mechanisms require further investigation.

We suggest regulation of LGMN by CST6 may be involved in placental development and function through extracellular matrix and tissue remodelling. In preeclampsia, low levels of LGMN and further inhibition of LGMN by elevated levels of CST6 may disrupt placental development. Furthermore, localised expression of CST6 and LGMN, observed in syncytiotrophoblast and EVT cells, further supports the potential role of these molecules in regulating trophoblast invasion and placental remodelling. More studies are required to confirm this.

Preeclampsia is associated with endothelial dysfunction^56^ and our data suggested combining CST6 with inflammatory cytokine TNFα may enhance endothelial dysfunction. This suggests that high circulating CST6 may exacerbate the maternal endothelial dysfunction observed in preeclampsia. Further studies are needed to elucidate the mechanisms behind this finding.

A significant strength of this study in the use of well characterised patient cohorts to confirm the dysregulation of CST6 and LGMN in preeclampsia. However, limitations to this study include the need to further investigate the role of CST6 and LGMN in trophoblast differentiation and function. Additionally, given their potential roles in EVT function, future studies should investigate the effects of inflammation and hypoxia on EVT cells and CST6 and LGMN expression.

Overall, our findings demonstrate that CST6 and LGMN are dysregulated in preeclampsia. To the best of our knowledge, this is the first description of CST6 and LGMN in human pregnancy pathology.

## Methods

### Samples from pregnancies complicated by early-onset preeclampsia (< 34 weeks’ gestation)

To characterise the expression of CST6 and LGMN in maternal plasma and placenta, ethics approval was granted by Mercy Health Human Research Ethics Committee (R11/34) and participants presenting to the Mercy Hospital for Women (Melbourne, Australia) gave informed, written consent for sample collection. Diagnosis of early-onset preeclampsia was conducted in accordance with the American College of Obstetricians and Gynaecologists (ACOG) guidelines^57^.

For messenger ribonucleic acid (mRNA) analysis, a total of 108 placental tissue samples were analysed comprising 78 participants with early-onset preeclampsia and 30 gestation-matched controls. Patient characteristics are shown in Table S1. All delivered via caesarean section. Control samples were obtained from women who were delivered preterm due to other complications such as placenta previa or spontaneous preterm rupture of membranes. Samples were excluded from both case and control groups if they exhibited congenital abnormalities or congenital infection confirmed through histopathological examination. Placental tissue samples were collected and processed within 30 min following delivery. Samples were processed and collected as per previously established methods^58^. Samples were then snap frozen and stored at − 80 °C for subsequent RNA extraction.

A total of 62 plasma samples were analysed. 35 participants had early-onset preeclampsia and 27 were normotensive gestation-matched controls who delivered at term without preeclampsia. Patient characteristics are shown in Table S2. Whole blood was collected in a 9 mL ethylenediaminetetraacetic acid (EDTA) BD Vacutainer^®^ K2E tube. Plasma was aliquoted and stored at − 80 °C until analysis.

### Biomarker and ultrasound measures for preventable stillbirth (BUMPS) − 36 weeks’ gestation predictive cohort

To evaluate the potential of CST6 and LGMN as a predictive biomarker, the Biomarker and Ultrasound Measures for Preventable Stillbirth (BUMPS) cohort was used^59^. This large-scale prospective study, conducted at Mercy Hospital for Women, involved collecting samples from a general, unselected pregnant population at 36 weeks of gestation. Ethical approval was obtained from the Mercy Health Human Research Ethics Committee (approval number 2019-012). Following written informed consent, women aged over 18 years, with a singleton pregnancy and normal mid-trimester fetal morphology examination were eligible to participate. Whole blood was collected in 9 mL EDTA tubes. Plasma supernatant was obtained and stored at − 80 °C until sample analysis.

The case-cohort of 205 samples were selected from the first 1 000 participants enrolled in the BUMPS study. This included all 21 women who delivered with preeclampsia, defined according to established ACOG guidelines^57^ and the control cohort comprised 184 women who delivered without preeclampsia. Detailed characteristics of the study participants are presented in Table S3.

### Culture of human trophoblast stem cells (hTSCs)

First-trimester hTSCs (CT30, female) were obtained from the RIKEN BRC through the National BioResource Project of the MEXT/AMED, Japan (RCB Cat#RCB4938, RRID: CVCL_A7BB) described in detail and cultured according to protocols outlined by Okae et al., 2018^26^.

### Differentiation of hTSCs to extravillous trophoblasts (EVTs) or syncytiotrophoblast cells [ST(2D)

Differentiation of hTSCs was conducted to determine the placental cell type expressing CST6 and LGMN. 24-well plates were pre-coated in iMatrix-511 (NovaChem Pty Ltd), incubated and washed as part of the hTSC protocol^26^.

For extravillous trophoblasts (EVTs) differentiation, once cells were washed, 60,000 cells/well were plated into 0.5 mL/well of syncytial EVT media respectively^26^. Cells were incubated at 37 °C for 10 min before resuspending cells and adding Matrigel^®^ (Bio-Strategy, Melbourne, Australia) to a final concentration of 2%. 48 h post-differentiation, cell lysates were collected for mRNA extraction and media was replaced with EVT medium without NRG1 prior to resuspension and addition of 2% Matrigel. Final lysates were collected at 96 h post-differentiation. Over the course of 96 h, morphological changes and alterations in established differentiation markers were monitored to confirm successful differentiation.

For syncytiotrophoblast cells [ST(2D)] differentiation, once cells were washed, 60,000 cells/well plated into 0.5 mL/well of syncytial [ST(2D)] media respectively^26^. Plated cells were incubated at 37 °C, 20% O_2_ and 5% CO_2_ until complete syncytialisation which was confirmed with morphology and changes to established differentiation markers across 96 h. Media was replaced with ST(2D) medium at 48 h post-differentiation.

### Placental explants

To characterise the expression of CST6 and LGMN in whole placental tissue, small pieces of villous tissue were cut from the mid-portion of the placenta to avoid the maternal and fetal surfaces. Tissues were washed with PBS and allowed to equilibrate to 37 °C for 1 h in DMEM (Life Technologies) containing 1% anti and 10% fetal calf serum. They were then dissected into small fragments of 1–2 mm size and four pieces put into each well of a 24 well plate. Treatments were administered following 24 h for either 24–48 h depending on the treatment condition.

### Hypoxic stimulation

hTSCs were differentiated into syncytiotrophoblast cells as described previously. Syncytialised hTSCs or placental explants were incubated in different oxygen concentrations. Cells or explants incubated in 8% oxygen, normoxic conditions, were incubated at 37 °C for 24 h or 48 h respectively. Hypoxia involved exposure to 1% oxygen for the same duration as previously described^58,60^. The media was collected for subsequent analysis and samples prepped for RNA extraction.

### Isolation of primary human umbilical vein endothelial cells (HUVECs)

To characterise the expression of CST6 and LGMN in endothelial cells, Human umbilical vein endothelial cells (HUVECs) were isolated from umbilical cord samples as previously described^61–63^. The umbilical cord was perfused with collagenase solution (1 mg/mL) (Worthington, Lakewood, NJ) within 30 min of delivery. HUVECs were cultured in M199 medium (Life Technologies) supplemented with 20% fetal calf serum, 1% antibiotic-antimycotic solution (Life Technologies), 1% endothelial cell growth factor (Sigma), and 1% heparin. Cells were plated at 10,000/cm^2^ and treated at 80% confluency. Importantly, only HUVECs between passages 2 and 4 were used in the experiments. The treatment solutions were prepared in 10% fetal calf serum (Sigma) and administered to the cells for a 24-hour period.

### Treatment of primary HUVECs with TNFα to induce endothelial dysfunction

HUVECs were plated at 22,500 cells/well in a 48-well tissue culture plate in HUVEC medium (M199 medium [Life Technologies] supplemented with 20% fetal calf serum, 1% antibiotic-antimycotic solution [Life Technologies], 1% endothelial cell growth factor [Sigma], and 1% heparin) for 24 h. Cells were then washed with PBS and treated with increasing doses of TNFα (Life Technologies, Carlsbad, CA, USA) at 0, 1 ng/mL and 10 ng/mL in 10% HUVEC media (M199 medium [Life Technologies] supplemented with 10% fetal calf serum, 1% antibiotic-antimycotic solution [Life Technologies], 1% endothelial cell growth factor [Sigma], and 1% heparin) for 24 h to induce endothelial dysfunction as previously described^61–63^. Conditioned media and cell lysates were collected for subsequent analysis.

### Treatment of primary HUVECs with Recombinant CST6

To obtain an appropriate treatment concentration of CST6, several optimisation experiments (data not shown) were conducted. These experiments were conducted using a range of concentrations with reference to the physiological plasma concentrations of CST6 observed in control and preeclamptic pregnancy samples from our established disease cohort.

HUVECs were plated as previously described. Cells were then washed with PBS and treated with vehicle control (PBS), TNFα (Life Technologies, Carlsbad, CA, USA) at 1 ng/mL, recombinant CST6 (R and D Systems, catalog no. 1286-PI-010) at 50 ng/mL or TNFα (1 ng/mL) + recombinant CST6 (50 ng/mL) for 24 h. Conditioned media and cell lysates were collected for subsequent analysis.

### Ribonucleic acid (RNA) extraction

RNA was isolated from placental samples and cell culture lines with the RNeasy^®^ Mini Kit (Qiagen) according to the manufacturer’s protocol. Once extracted, RNA was quantified with a Nanodrop ND 1000 spectrophotometer (NanoDrop Technologies Inc, Wilmington, DE, USA).

### Reverse transcription to generate complementary deoxyribonucleic acid (cDNA)

RNA extracted from cells and tissues was converted to complementary deoxyribonucleic acid (cDNA) using the Applied Biosystems high-capacity cDNA reverse transcriptase kit (Life Technologies, Carlsbad, USA) according to manufacturer’s instructions. Equal amounts of RNA were reverse transcribed into cDNA on the Applied Biosystems MiniAmp Thermal Cycler (ThermoFisher Scientific) following the manufacturer’s instructions. The conversion was performed under the following conditions: 25 °C for 10 min, 37 °C for 60 min and 85 °C for 5 min. cDNA was stored at -20 °C prior to processing for quantitative reverse transcriptase polymerase chain reaction (RT-qPCR).

### Quantitative reverse transcriptase polymerase chain reaction (RT-qPCR)

RT-qPCR was conducted to quantify mRNA expression levels of the following genes: *e-cadherin 1* (*CDH1*, Hs01023895_m1), *GATA binding protein 3* (*GATA3*, Hs00231122_m1), *human leukocyte antigen G* (*HLA-G*, Hs03045108_m1), *syndecan-1* (*SDC1*, Hs00896423_m1), *TEA domain transcription factor 4* (*TEAD4*, Hs01125032_m1), *vascular cell adhesion protein 1* (*VCAM-1*,* Hs01003372_m1*), *Endothelin 1* (*ET-1*,* Hs00174961_m1*), *intercellular Adhesion Molecule 1* (*ICAM-1*,* Hs00164932_m1*), legumain (*LGMN*, Hs00271599_m1) and *cystatin E/M* (*CST6*,* Hs00154599_m1*) (all Life Technologies), on a CFX384 (BioRad) machine or Applied Biosystem QuantStudio 5 (ThermoFisher Scientific). The master mix included FAM-labelled Taqman™ Fast Advanced Master Mix (ThermoFisher Scientific) and their respective primer (Life Technologies). The run conditions used were as follows: 95 °C for 20s, (95 °C for 3s and 60 °C for 30s) x 40 cycles.

All data were normalised to their respective housekeeper genes, depending on the conditions and cell/tissue type: *Tyrosine 3-Monooxygenase/Tryptophan 5-Monooxygenase Activation Protein Zeta* (*YWHAZ*, Hs01122454_m1) was used for HUVEC or in vitro experiments, *glyceraldehyde 3-phosphate dehydrogenase* (*GAPDH*,* Hs99999905_m1*) was used for hTSC differentiation to syncytiotrophoblast cells or the geometric mean of *Topoisomerase-1* (*TOP1*, Hs00243257_m1) and *Cyclin-1* (*CYC1*, Hs00357717_m1) which was used for placental samples or for in vitro experiments. *CYC1* was also used for hTSC differentiation to EVTs. Each cDNA sample was run in duplicate for RT-qPCR analysis, and the average cycle threshold (Ct) value was used for further calculations. To account for variations, gene expression was normalized to the mean Ct value obtained from the control group for each experiment. The 2^−ΔΔCt^ method was used to quantify the relative fold change in gene expression compared to the controls.

### Protein extraction

To isolate protein from placental tissue, a RIPA buffer containing a protease inhibitor cocktail (Sigma-Aldrich; St. Louis, MO, USA) and Halt™ phosphatase inhibitor cocktail (Thermo Fisher Scientific; Waltham, MA, USA) was used to lyse cells. Centrifugation was used to pellet cellular debris and to remove the protein from the lysate solution. The Pierce™ BCA assay (Thermo Fisher Scientific) was performed according to the manufacturer’s protocol to quantify protein in each sample. Equal protein amounts were loaded for ELISA.

### Enzyme linked immunosorbent assays (ELISAs)

CST6 and LGMN protein levels were measured in maternal plasma samples and placental lysates via ELISA. The large cohort analyses were analysed using an Invitrogen Human Cystatin E/M Sandwich ELISA kit (Thermo Fischer Scientific, catalog no. EH142RB) and a R&D Systems Human Total Legumain DuoSet ELISA kit (R and D Systems, catalog no. RDSDY4769), following manufacturer’s specifications. The < 34 week and BUMPS plasma samples were diluted 1:8 for CST6 and 1:5 for LGMN, following optimisation. Placental lysates were run neat.

### scRNA-seq analysis of a publicly available hTSC-derived organoid model for trophoblast differentiation

To analyse the scRNA-seq data, the code previously published (https://github.com/MatthewJShannon) was applied on the dataset (GEO accession number GSE174481) for the pre-processing steps and adjusted for subsequent analysis^27^. For this analysis, the detailed methodology has been previously described using the same pipeline and dataset^30^.

Characteristic gene markers for each cell type were used to annotate clusters. Each cluster was annotated and resulted in 6 distinct transcriptomic cell identities: cytotrophoblast (CTB), proliferative cytotrophoblast (CTBprol), pre-fusion cytotrophoblast (CTBpf), progenitor EVT (pEVT), invasive EVT (iEVT) and syncytiotrophoblast (STB)^64–69^. A dot plot including the marker genes and general trophoblast markers (*EGFR*, *KRT7*,* TFPA2C*,* GATA3*) is shown in the Supplementary Fig. 1^70^. Dot plot and feature plots were generated for the genes *CST6* and *LGMN* to visualize their respective expression. The full code is available upon request.

### Adapted snRNA-seq analysis of placental samples from various pregnancies

snRNA-seq analysis was used to validate specific cell type expression of CST6 and LGMN in the placenta of both early-onset preeclampsia and controls. We adjusted the analysis from the protocol and dataset outlined in the manuscript by Nonn, et al., (EGA accession number: EGAS00001005681 and processed data from original manuscript available at: https://zenodo.org/records/8159511 upon request)^28^. The code for reproducing visualisations and analysis is available at https://github.com/HiDiHlabs/preeclamspsia_Nonn_etal/.

In this study uteroplacental tissue was sampled at 5 to 11 weeks for the preterm control cohort, 27 to 33 weeks of gestation for the early-onset preeclampsia cohort and at 38 to 40 weeks for healthy term control cohort. It was not possible to collect placentas from healthy controls at the same gestational time point as the preeclampsia cohort. Therefore, there was an adjustment for potential confounding of a preterm control gene expression profile which would possibly interfere with the snRNA-seq expression profile of the early-onset preeclampsia cohort. Therefore, the authors integrated a published scRNA-seq data set that compared preterm births that were not a result of preeclampsia^28^.

In this analysis 21 placental samples were studied at various stages of pregnancy as described by Nonn, et al.^28^. We adjusted our analysis to focus only on the chorionic villi samples of the placenta. 79 885 nuclei of preterm control samples (*n* = 10), 29 702 of term control (healthy term pregnancy) samples (*n* = 6) and 36 050 nuclei of early-onset preeclampsia samples (*n* = 5) were sequenced and harmonised. The relevant quality controls and data pre-processing was conducted as per the methodology defined by Nonn, et al.^28^.

### Statistical analysis

Maternal characteristics were compared for women diagnosed with preeclampsia, compared to normotensive, gestation-matched controls using a Mann–Whitney U test for continuous data and Fisher’s exact test for categorical data. The data obtained was tested for normality using the Anderson–Darling test, D’Agostino & Pearson test, Shapiro–Wilk test, and Kolmogrov-Smirnov test. For two unpaired groups, an unpaired t-test (parametric) or Mann–Whitney test (non-parametric) test was used. For analysis of two paired groups, a paired t-test (parametric) or a Wilcoxon ranked test (non-parametric) was used. For ≥ 3 groups, either one-way ANOVA (parametric) or a Kruskal Wallis test (non-parametric) was used. Parametric data was represented as mean ± standard error of the mean (SEM) while non-parametric data was represented as a median with interquartile range (IQR). *P* < 0.05 were considered statistically significant. All in vitro experiments were performed in technical duplicates or triplicates and repeated at least five times unless stated otherwise. All analyses were performed using GraphPad Prism 10.2.3 (GraphPad Software, LLC.).

## Electronic supplementary material

Below is the link to the electronic supplementary material.

Supplementary Material 1

## Data Availability

The data generated and/or analysed during this study are included in this manuscript and within the supplementary file. Data and resources used in this study are available from the corresponding author upon reasonable request. The full code used for the scRNA-seq and snRNA-seq analysis is available upon reasonable request. Data used in this study for snRNA-seq analysis is available at EGA accession number: EGAS00001005681 and processed data from original manuscript available at: https://zenodo.org/records/8159511 upon request from the authors. Data for the scRNA-seq analysis is available at GEO accession number: GSE174481.
