# Supplementary material for: Cystatin 6 (CST6) and Legumain (LGMN) are potential mediators in the pathogenesis of preeclampsia
Source: Sci Rep. 2025 Apr 15;15:12945. doi: 10.1038/s41598-025-96823-9 (PMC12000359; doi:10.1038/s41598-025-96823-9)
Supplement: Supplementary file 1 — Supplementary Material 1 [file 41598_2025_96823_MOESM1_ESM.pdf]

Supplementary information

Cystatin 6 (CST6) and Legumain (LGMN) are potential mediators in the pathogenesis of preeclampsia.

Authors:

Stefan M. Botha[1,2,3,4,5,6, *], Lucy. A. Bartho[1,2], Sunhild Hartmann[1,2,3,4,5,6], Ping Cannon[1,2], Anna Nguyen[1,2], Tuong-Vi Nguyen[1,2], Natasha Pritchard[2], Ralf Dechend[3,4,5,6,7], Olivia Nonn[3,4,5,6], Stephen Tong[1,2], Tu'uhevaha J. Kaitu'u-Lino[1,2]

Affiliations:

[1] Translational Obstetrics Group, The Department of Obstetrics, Gynaecology and Newborn Health, Mercy Hospital for Women, University of Melbourne, Heidelberg, Victoria, 3084, Australia

[2] Mercy Perinatal, Mercy Hospital for Women, Heidelberg, Victoria, Australia

[3] Charité – Universitätsmedizin Berlin, corporate member of Freie Universität Berlin and Humboldt-Universität zu Berlin, Berlin, Germany

[4] Experimental and Clinical Research Center, a cooperation between the Max-Delbrück-Center for Molecular Medicine in the Helmholtz Association and the Charité-Universitätsmedizin Berlin, Berlin, Germany

[5] Max-Delbrück-Center for Molecular Medicine in the Helmholtz Association (MDC), Berlin, Germany

[6] German Center for Cardiovascular Research (DZHK), partner site Berlin, Germany

[7] HELIOS Clinic, Department of Cardiology and Nephrology, Berlin, Germany

**Corresponding author: bothas@student.unimelb.edu.au*

Table S1: Patient characteristics for CST6 and LGMN mRNA analysis of placental tissue samples from pregnancies complicated by early-onset preeclampsia (< 34 weeks' gestation). Abbreviations: BMI - Body mass index, SBP - Systolic blood pressure and DBP - Diastolic blood pressure. Mann-Whitney U tests used for comparison of medians. Chi-square tests used for categorical variables. BMI data missing for 8/30 control samples and 12/78 preeclampsia samples. Birthweight, SBP and DBP data missing for 1/78 preeclampsia samples. Unpaired t-test was used for comparison of means (for normally distributed data), Mann-Whitney U tests for medians (if not normally distributed), and Chi-square tests for categorical variables.

	Control (*n = 30*)	Preeclampsia (*n = 78)*	P-value (Control vs Preeclampsia)
**Maternal Age** (years)<br>Mean ± SEM	30 ± 1.09	30.54 ± 0.62	0.691
**Gestation at Delivery** (weeks)<br>Mean ± SEM	30.05 ± 0.44	29.64 ± 0.28	0.449
**BMI** (kg/m2)<br>Median (IQR)	28.2 (45 – 19)	27.05 (50 – 18)	0.580
**Parity no.** (%)			0.051
0	13 (43.33)	53 (67.95)	
1	10 (33.33)	17 (21.79)	
≥ 2	7 (23.33)	8 (10.26)	
**SBP at Delivery** (mmHg)<br>Median (IQR) ****	120 (135 – 105)	170 (220 – 140)	< 0.0001
**DBP at Delivery** (mmHg)<br>Median (IQR) ****	71.5 (85 – 55)	105 (130 – 80)	< 0.0001
**Birth weight** (g)<br>Median (IQR) ***	1572 (2274 – 674)	1127 (2637 – 420)	0.0001
**Assigned female at birth** no. (%)	12 (40)	39 (50)	0.351

Table S2: Patient characteristics for analysis of circulating CST6 and LGMN from pregnancies complicated by early-onset preeclampsia (< 34 weeks' gestation). Abbreviations: BMI - Body mass index, SBP - Systolic blood pressure and DBP - Diastolic blood pressure. Mann-Whitney U tests used for comparison of medians. Chi-square tests used for categorical variables. BMI data missing for 3/35 preeclampsia samples. Birthweight, SBP and DBP data missing for 1/27 control samples, and 1/35 preeclampsia samples. Unpaired t-test was used for comparison of means (for normally distributed data), Mann-Whitney U tests for medians (if not normally distributed), and Chi-square tests for categorical variables.

	Control (*n = 27*)	Preeclampsia (*n = 35)*	P-value (Control vs Preeclampsia)
**Maternal Age** (years) Mean ± SEM	32.37 ± 0.67	30.14 ± 1.32	0.142
**Gestation at Delivery** (weeks) **** Mean ± SEM	37.68 ± 1.46	29.96 ± 0.50	< 0.0001
**Gestation at blood collection** (weeks) Mean ± SEM	27.76 ± 0.96	29.86 ± 0.45	0.259
**BMI** (kg/m2) Median (IQR) **	25 (44.2 – 19.1)	28.7 (50.2 – 20)	0.0025
**Parity no.** (%)			0.1431
0	13 (48.15)	25 (71.43)	
1	8 (29.63)	7 (20)	
≥ 2	6 (22.22)	3 (8.57)	
**SBP at Delivery** (mmHg) Median (IQR) ****	120 (130 – 90)	170 (220 – 150)	< 0.0001
**DBP at Delivery** (mmHg) Median (IQR) ****	75 (85 – 60)	100 (130 – 90)	< 0.0001
**Birth weight** (g) Median (IQR) ****	3445 (4870 – 2830)	1284 (2960 – 431)	< 0.0001
**Assigned female at birth** no. (%)	10 (37.04)	17 (48.57)	0.3113

Table S3: Patient characteristics for analysis of circulating CST6 and LGMN in patients preceding diagnosis of term preeclampsia (36 weeks). Abbreviations: BMI - Body mass index. Mann-Whitney U tests used for comparison of medians. Chi-square tests used for categorical variables. BMI data missing for 1/182 control samples. Gestation at delivery data missing for 51/182 control samples and 15/21 preeclampsia samples. Unpaired t-test was used for comparison of means (for normally distributed data), Mann-Whitney U tests for medians (if not normally distributed), and Chi-square tests for categorical variables.

	**Control (*n = 184*)**	**Preeclampsia (*n = 21)***	**P-value (Control vs Preeclampsia)**
**Maternal Age** (years) Mean ± SEM *	32.11 ± 0.30	33.67 ± 1.03	0.1181
**Gestation at Delivery** (weeks) Mean ± SEM ****	39.60 ± 0.10	37.30 ± 0.52	< 0.0001
**Gestation at blood collection** (weeks) Median (IQR)	36.14 (37.14 – 34.85)	36.28 (37.28 – 34.85)	0.2694
**BMI** (kg/m2) Median (IQR) *	24.55 (45.53 – 17.99)	27.88 (36.06 – 20.24)	0.026
**Parity no.** (%) **			0.0012
0	89 (48.37)	19 (90.48)	
1	74 (40.22)	2 (9.52)	
≥ 2	21 (11.41)	0 (0.00)	
**Birth weight** (g) Median (IQR) ***	3480 (4470 – 2460)	3120 (4094 – 2230)	0.0005
**Assigned female at birth** no. (%)	93 (50.54)	10 (47.62)	0.7995

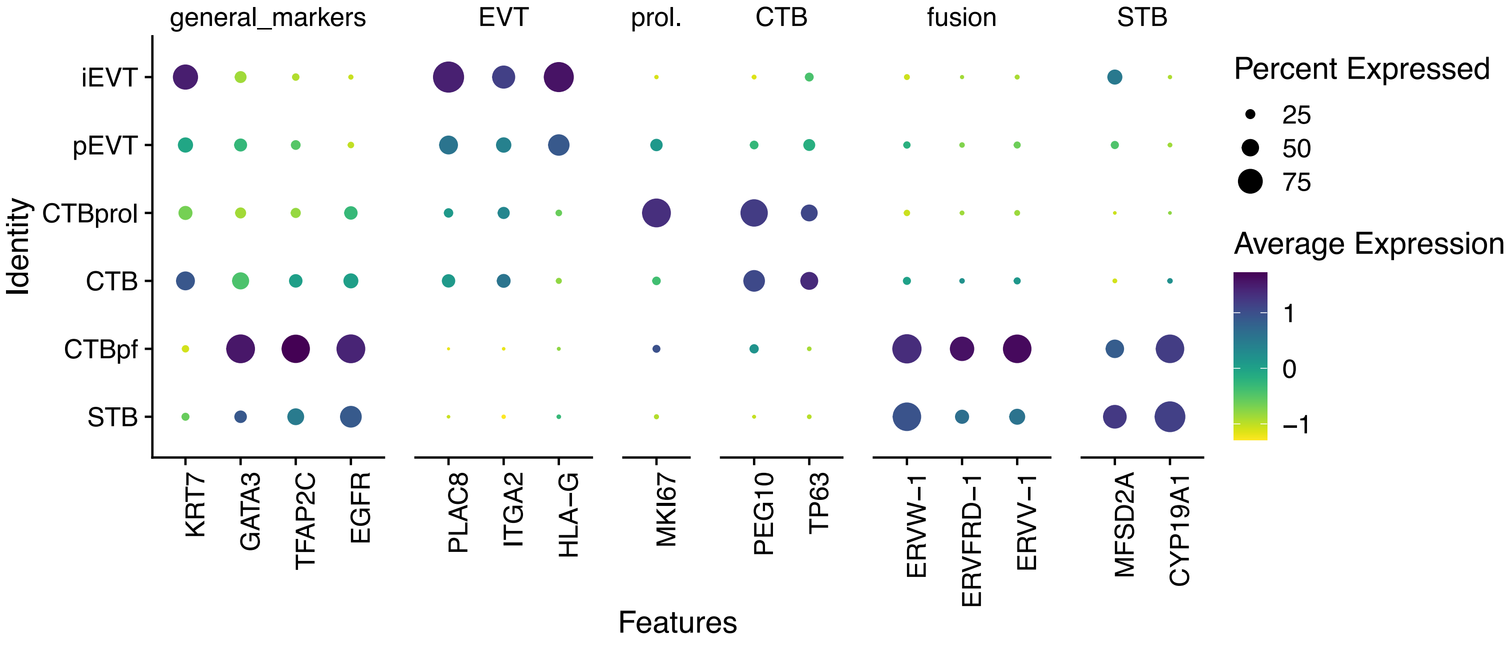

Supplementary figure 1. Dot plot of markers used for cell identities in scRNA-seq analysis. *Transcriptomic analysis performed on a publicly available scRNA-seq dataset of n=3 three-dimensional human trophoblast stem cell (hTSC)-derived organoids treated under hTSC conditions or induced to differentiate to extravillous trophoblasts (EVTs) for 21 days [27]. 6 transcriptomic cell states were identified for analysis of CST6 and LGMN expression in each cell culture condition: cytotrophoblast (CTB), proliferative cytotrophoblast (CTBprol), pre-fusion cytotrophoblast (CTBpf), progenitor EVT (pEVT), invasive EVT (iEVT) and syncytiotrophoblast (STB).*

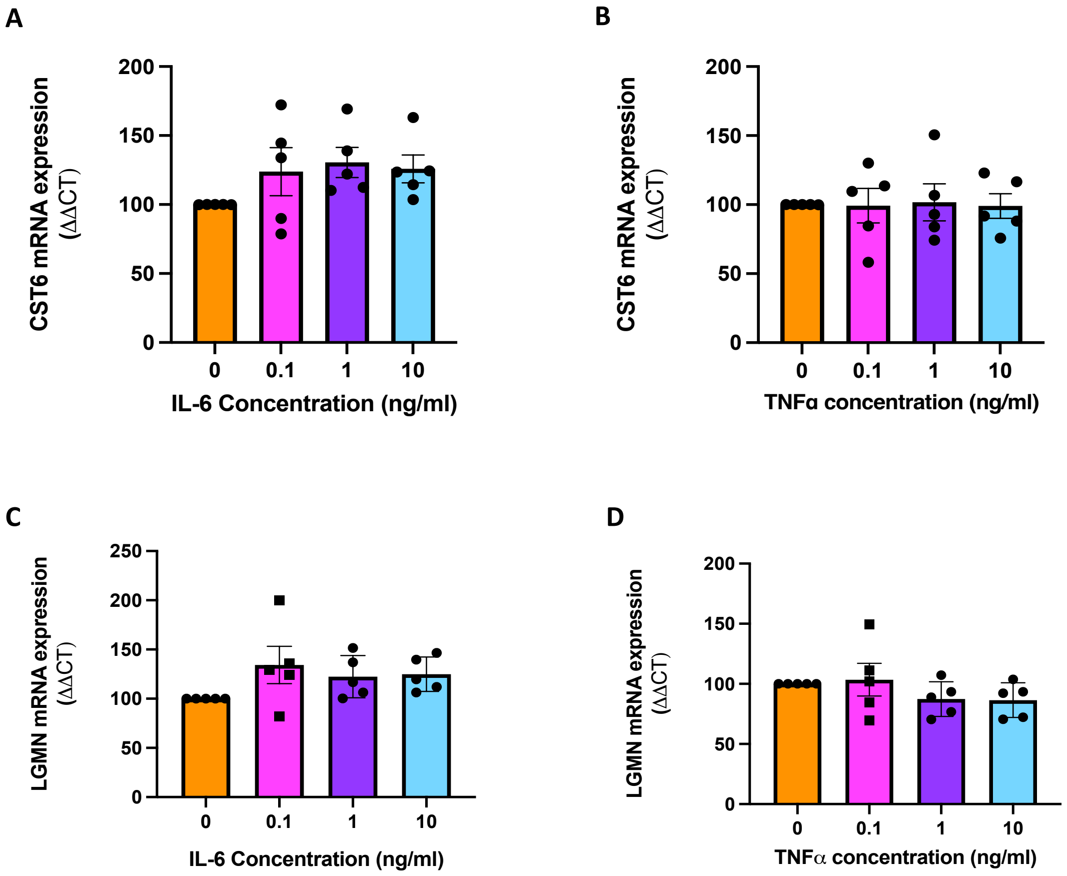

Supplementary figure 2: Regulation of CST6 and LGMN expression in syncytiotrophoblast cells. *CST6 and LGMN expression was unchanged following treatment with increased doses of* ***(A), (C)*** *IL-6 and* ***(B), (D)*** *TNFα. mRNA expression was normalised to the geometric mean of housekeeper genes or single housekeeping gene depending on the treatment. To calculate significance, one-way ANOVA or Unpaired t test (parametric) or a Kruskal Wallis test (non-parametric) was used. Data expressed as mean ± SEM for experiments. Experiments repeated n=5 in triplicate.*

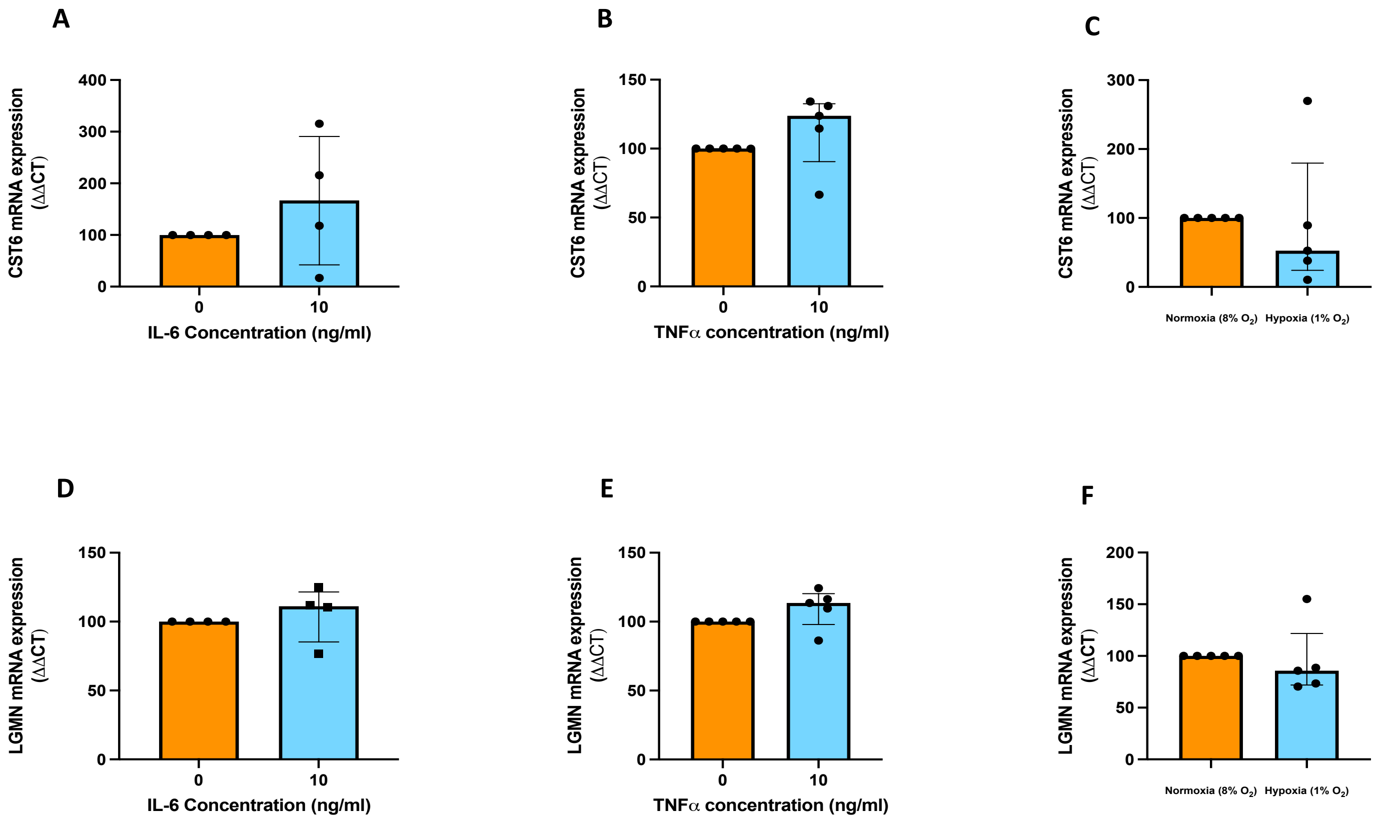

Supplementary figure 3: Regulation of CST6 and LGMN expression in placental explants. *CST6 expression was unchanged in placental explants treated with* ***(A)*** *IL-6 and* ***(B)*** *TNFα.* ***(C)*** *For CST6 mRNA expression during hypoxia, the same was observed in placental explants incubated in a hypoxic environment (1% O_2) compared to control normoxic environment (8% O_2). LGMN expression was unchanged in placental explants treated with* ***(D)*** *IL-6 and* ***(E)*** *TNFα.* ***(F)*** *For LGMN mRNA expression during hypoxia, the same was observed in placental explants incubated in a hypoxic environment (1% O_2) compared to control normoxic environment (8% O_2). mRNA expression was normalised to the geometric mean of housekeeper genes or single housekeeping gene depending on the treatment. To calculate significance, one-way ANOVA or Unpaired t test (parametric) or a Kruskal Wallis test (non-parametric) was used. Data expressed as mean ± SEM. Experiments repeated n=5 in triplicate.*
